# Motif clustering and digital biomarker extraction for free-living physical activity analysis

**DOI:** 10.1186/s13040-025-00424-1

**Published:** 2025-01-22

**Authors:** Ya-Ting Liang, Charlotte Wang

**Affiliations:** 1 Institute of Epidemiology and Preventive Medicine, College of Public Health, National Taiwan University, Taipei, Taiwan; 2 Institute of Health Data Analytics and Statistics, College of Public Health, National Taiwan University, No. 17, Xu-Zhou Road, Taipei, 100025 Taiwan; 3 Master of Public Health Program, College of Public Health, National Taiwan University, Taipei, Taiwan

**Keywords:** Digital biomarker, Activity pattern, Functional data analysis, Feature extraction, Clustering, Association, Wearable device data

## Abstract

**Background:**

Analyzing free-living physical activity (PA) data presents challenges due to variability in daily routines and the lack of activity labels. Traditional approaches often rely on summary statistics, which may not capture the nuances of individual activity patterns. To address these limitations and advance our understanding of the relationship between PA patterns and health outcomes, we propose a novel motif clustering algorithm that identifies and characterizes specific PA patterns.

**Methods:**

This paper proposes an elastic distance-based motif clustering algorithm for identifying specific PA patterns (motifs) in free-living PA data. The algorithm segments long-term PA curves into short-term segments and utilizes elastic shape analysis to measure the similarity between activity segments. This enables the discovery of recurring motifs through pattern clustering. Then, functional principal component analysis (FPCA) is then used to extract digital biomarkers from each motif. These digital biomarkers can subsequently be used to explore the relationship between PA and health outcomes of interest.

**Results:**

We demonstrate the efficacy of our method through three real-world applications. Results show that digital biomarkers derived from these motifs effectively capture the association between PA patterns and disease outcomes, improving the accuracy of patient classification.

**Conclusions:**

This study introduced a novel approach to analyzing free-living PA data by identifying and characterizing specific activity patterns (motifs). The derived digital biomarkers provide a more nuanced understanding of PA and its impact on health, with potential applications in personalized health assessment and disease detection, offering a promising future for healthcare.

**Supplementary Information:**

The online version contains supplementary material available at 10.1186/s13040-025-00424-1.

## Introduction

The World Health Organization (WHO) defines physical activity (PA) as any bodily movement that expends energy. This encompasses various activities, from daily chores to recreational pursuits [1, 2]. Recognizing the crucial role of PA in maintaining health, the WHO emphasizes the importance of regular exercise [1]. Growing research has investigated its impact on health-related issues, including its association with disease [3–5], its role in specific populations [6, 7], its influence on sleep [8, 9] and rest-activity rhythms [10, 11], and its significance in disease management [5] and prevention [5, 12].

As a result of advancements in technology and the development and popularization of wearable devices, many researchers have begun to collect real-world PA data through wearable devices and apply this data to biomedical and health-related research [13]. However, free-living PA data from wearable devices lacks activity-type labels. Consequently, the data can only provide information on the overall intensity of an individual’s daily activity levels but cannot accurately identify specific activity types. Hence, in biomedical and health-related research, researchers often calculate units of PA intensity (such as activity count, ENMO [14], signal vector magnitude (SVM) [15, 16], signal magnitude area (SMA) [16], physical activity index [17], and Anglez [18]), and then derive summary statistics, such as time domain measures (e.g., mean, variance) and frequency domain measures (e.g., magnitude, dominant frequency), or other activity metrics (such as moderate-to-vigorous physical activity (MVPA) [19, 20]) over fixed time intervals (e.g., daily, weekly). These metrics, calculated either on the merged triaxial PA data or on each axis independently, are typically used as digital biomarkers and incorporated into models for analysis [15, 19–22]. Although sufficient for overall trends, such summary statistics are not designed to describe temporal patterns across time intervals that are relatively shorter than a day. Therefore, defining more informative digital biomarkers that could reveal these temporal details and represent daily specific PA patterns would enhance our understanding of the impact of particular activity behaviors on health (e.g., [23]). and improve the interpretability and applicability of research findings.

Cluster analysis is an exploratory data analysis technique that can uncover potential subgroups within data when there is no prior knowledge about the data or the underlying clustering structure. Hence, we propose a clustering algorithm to identify PA patterns in short fixed-time intervals and identify potential activity-type labels as digital biomarkers. This might resolve problems in data analysis and model building from inter-individual differences and could define more informative digital biomarkers rather than overall summary statistics.

Recent research has employed functional data analysis (FDA) methods to analyze wearable device data [21, 24, 25]. FDA treats time series data as functions, capturing variations and addressing measurement errors through smoothing techniques [24, 26, 27]. This approach can effectively address potential measurement errors associated with wearable device data while simultaneously capturing the time-varying trends of PA patterns. Hence, we integrate the proposed clustering algorithm and FDA to explore the patterns of free-living PA. This method addresses measurement errors introduced during data collection and incorporates temporal information. It also identifies effective digital biomarkers representing activity patterns to investigate the impact of activity pattern changes on health events.

As noted in Jacques et al.’s (2014) [28] comprehensive review of functional data clustering methods, these techniques primarily aim to identify groups of curves with similar overall shapes or patterns. Given the substantial inter-individual differences/variability in terms of the types and durations of daily activities among individuals, identifying specific activity segments or patterns within whole-day activity curves can provide valuable insights for uncovering unique activity phenomena. This information can potentially improve some clinical applications, enabling tailored interventions for promoting health and detecting early signs of disease.

Motifs, defined as recurring patterns within time series data, offer deep insights into the phenomena underlying the curves [29, 30]. In free-living PA data, motifs represent specific activity patterns, reflecting either common behaviors across individuals or recurrent activity patterns particular to an individual. These motifs offer valuable digital biomarkers, providing insights into population-level trends and individual-specific behaviors. By identifying motifs, researchers can better understand the relationship between PA and health, enabling the development of more effective health promotion strategies. Furthermore, research has shown that specific activity patterns like gait are linked to health outcomes like Alzheimer’s disease [23]. This finding underscores the potential for identifying motifs associated with specific health conditions, informing the development of targeted interventions and preventative strategies. Given these considerations, our proposed clustering method explicitly targets identifying motifs in free-living PA data. These motifs can then be leveraged to define digital biomarkers to analyze health-related events.

In summary, this study proposes an elastic distance-based motif clustering algorithm for identifying motifs in free-living PA data and then utilizing the functional principal component analysis to define digital biomarkers from each motif. Finally, association and classification models are constructed based on these digital biomarkers for advancing personalized health assessment and disease detection.

## Methods

### Elastic distance-based motif clustering and digital biomarker identification

#### Elastic shape analysis

Functional data typically exhibit two types of variation: phase variation and amplitude variation. Phase variation refers to the variability in the timing or temporal shift of similar waveforms, while amplitude variation refers to differences in the intensity of PA [31]. Distances used in traditional functional clustering methods, like Euclidean distance and dynamic time warping (DTW), primarily focus on comparing point-to-point curve amplitude, lacking the ability to effectively capture phase differences [32]. DTW minimizes a penalized $\:{\mathbb{L}}^{2}$ norm, often resulting in suboptimal alignment [33]. Wasserstein distance, while providing a distribution-based measure, requires substantial data [34]. Elastic shape analysis employs a phase-amplitude separation procedure to register functional data, enabling statistical analyses of the distinct phase and amplitude components [33, 35–38]. The elastic distance is a metric with both phase and amplitude variability [35, 38, 39]. Avoiding the pinching effect inherent in alignment [33], elastic distance is better suited for analyzing free-living PA curves where both phase and amplitude variations are significant.

Elastic shape analysis employs the square root velocity function (SRVF) framework for curve registration [35, 37, 38]. Let $\:f\::\:[0,\:1]\:\to\:\:{R}^{n}$ be a Euclidean curve. Srivastava et al. (2011) [37] established a mathematical expression representing a function $\:f$ using its SRVF and defined it as

1 $$\:q\left(t\right)\equiv\:\frac{\dot{f\left(t\right)}}{\sqrt{\left|\dot{f\left(t\right)}\right|}}$$

where $\:\dot{f\left(t\right)}$ is the velocity vector at $\:f\left(t\right)$. If we warped a function $\:f$ by $\:\gamma\::f\to\:\left(f\circ\:\gamma\:\right)$, the SRVF of $\:(f\circ\:\gamma\:)$ is $\:(q\circ\:\gamma\:)\sqrt{\dot{\gamma\:}}$ according to the chain rule. Then, the distance between two functions can be defined as

2 $$\:d\left({f}_{1},{f}_{2}\right)=d\left({f}_{1}\circ\:\gamma\:,{f}_{2}\circ\:\gamma\:\right)=||\left({q}_{1}\circ\:\gamma\:\right)\sqrt{\dot{\gamma\:}}-\:\left({q}_{2}\circ\:\gamma\:\right)\sqrt{\dot{\gamma\:}}||=||{q}_{1}-{q}_{2}||$$

where $\:||\bullet\:||$ is the standard $\:{\mathbb{L}}^{2}$ norm [37]. Subsequently, the amplitude and phase distance between the two functions can be calculated as follows:

3 $$\:{d}_{amp}\left({f}_{1},{f}_{2}\right)=||{q}_{{f}_{1}\circ\:l}-{q}_{{f}_{2}\circ\:\gamma\:}||$$,

4 $$[d_{phs}(f_1, f_2) = \cos^{-1} \left( \int_0^1 \sqrt{{l}\dot(t)} \sqrt{{\gamma}\dot(t)} \, dt \right)= \cos^{-1} \left( \int_0^1 \sqrt{1} \sqrt{{\gamma}\dot(t)} \, dt \right)= \cos^{-1} \left( \int_0^1 \sqrt{{\gamma}\dot(t)} \, dt \right)$$

where $\:\gamma\:$ is the warping function aligning $\:{f}_{2}$ to $\:{f}_{1}$ and $\:l\left(t\right)=t$ [37, 38] (for more details, please refer to Srivastava and Klassen (2016) [38]). By computing the elastic distance, we can derive the phase and amplitude distance, enabling the measurement of the similarity between PA curves.

#### Motif clustering algorithm

PA data collected from wearable devices is typically multi-axial, resulting from measuring and reporting signal information by accelerometers and gyroscopes. Existing functional data clustering methods focus on analyzing unidimensional functions with limited attention to multidimensional functional data. Moreover, there is a dearth of literature proposing methods for clustering motifs. Consequently, this study proposes an elastic distance-based motif clustering approach tailored explicitly for multidimensional PA data. This method, employing elastic distance and the K-means clustering algorithm, allows for differential weighting of axes based on prior knowledge.

First, the long-term free-living PA curves are split into shorter time windows for analysis, such as 30-minute or 1-hour intervals. Let $\:{x}_{ij}\left(t\right)$ be the $\:j$th curve for the $\:i$th individual. The steps involved in the elastic distance-based motif clustering algorithm are as follows:

1. Partition long-term free-living PA curves into shorter-term activity epochs
2. Choose the number of clusters ($\:K$) and randomly assign each cluster’s centered function $\:{c}^{\left(1\right)}\left(t\right),\:{c}^{\left(2\right)}\left(t\right),\:\dots\:,\:{c}^{\left(K\right)}\left(t\right)$.
3. Calculate the elastic distance between each functional curve and each cluster center. Since the elastic distance contains phase and amplitude distance, we assign weights for these two distances to represent their importance in different application scenarios. The metric for one-axis PA data is displayed by the following formula:
  5 $$\:{d}_{elastic}\left({x}_{ij}\left(t\right),\:{c}^{\left(k\right)}\left(t\right)\right)={w}_{p}\times{d}_{phs}\left({x}_{ij}\left(t\right),\:{c}^{\left(k\right)}\left(t\right)\right)+{w}_{a}\times{d}_{amp}\left({x}_{ij}\left(t\right),\:{c}^{\left(k\right)}\left(t\right)\right)$$
  where $\:{d}_{amp}\left({x}_{ij}\left(t\right),\:{c}^{\left(k\right)}\left(t\right)\right)=||{q}_{{c}^{\left(k\right)}\left(t\right)\circ\:l}-{q}_{{x}_{ij}\left(t\right)\circ\:\gamma\:}||$, ${d_{phs}}({x_{ij}}(t),\left. {{c^{(k)}}(t)} \right) = {\cos ^{ - 1}}\left( {\int_0^1 {\sqrt {{\gamma} \dot(t)} dt} } \right)$, and $\:{w}_{p}+{w}_{a}=1$. This formula can be extended to multi-axis PA data ($\:h=\text{1,2}\dots\:,H$):
  6 $$\:{d}_{elastic}({x}_{ij}\left(t\right),\:{c}^{\left(k\right)}(t\left)\right)=\sum\:_{h}^{}\left\{{w}_{p,h}\times{d}_{phs}({x}_{ijh}\left(t\right),\:{c}_{h}^{\left(k\right)}(t\left)\right)+{w}_{a,h}\times{d}_{amp}({x}_{ijh}\left(t\right),\:{c}_{h}^{\left(k\right)}(t\left)\right)\right\}$$
  where $$\:\sum\:_{h}^{}\left({w}_{p,h}+{w}_{a,h}\right)=1$$.
4. Assign each function curve to the cluster with the shortest elastic distance
  7 $$\:{L}_{ij}=\underset{k}{\arg\min}{d}_{elastic}$$
  where $\:{L}_{ij}$ is the label for $\:{x}_{ij}\left(t\right).$
5. Recalculate the centered function for each cluster with
  8 $$\:{c}^{\left(k\right)}\left(t\right)=\frac{1}{\left|{S}_{k}\right|}\sum\:_{{x}_{ij}\left(t\right)\in\:{S}_{k}}{x}_{ij}\left(t\right)$$
  $\:{S}_{k}:\:$the set of activity function assigned to the cluster $\:k$,
  $\:\left|{S}_{k}\right|:\:$the number of activity function in the cluster $\:k.$
6. Repeat steps 3 to 5 until the stopping criteria are met. We can then obtain the clustering label for each functional curve.

### Identification of digital biomarkers through motif clustering

Considering each person has multiple recording days $\:(d=1,\:\dots\:,D)$, we can calculate the mean activity functions $\:{A}_{id}^{\left(k\right)}\left(t\right)$ for the $\:i$th individual on the $\:d$th day within the $\:k$th cluster to summarize people’s activity patterns. If there are no activity segments for a specific cluster on a given day, the activity mean function for that cluster would be 0. Then we can utilize these mean functions within each cluster to guide further analysis. Letting

9 $$\:{A}_{id}^{\left(k\right)}=\left\{\begin{array}{c}\frac{1}{\left|{S}_{id}^{\left(k\right)}\right|}\sum\:_{{x}_{ij}\left(t\right)\in\:{S}_{id}^{\left(k\right)}}{x}_{ij}^{\left(k\right)}\left(t\right)\\\:0\:,\:if\:\left|{S}_{id}^{\left(k\right)}\right|=0\:\end{array}\right.$$

where $\:{S}_{id}^{\left(k\right)}\:$ is the set of activity functions assigned to the cluster $\:k$ for the $\:i$th individual on the $\:d$th day and $\:\left|{S}_{id}^{\left(k\right)}\right|$ is the number of activity function in the cluster $\:k$ for the $\:i$th individual on the $\:d$th day.

Once the mean function of each person within each cluster is determined, the cluster-based digital biomarkers can be considered as features and used in statistical analysis. To investigate the dynamics of variable distributions, we adapt functional principal component analysis (FPCA) to describe activity patterns. The objective of FPCA is to identify a set of orthogonal components that can adequately explain the variance of observations with the fewest possible features [27].

Using the Karhunen–Loève decomposition, the mean activity function $\:{A}_{id}^{\left(k\right)}\left(t\right)$ can be represented in the following model:

10 $$\:{A}_{id}^{\left(k\right)}\left(t\right)={\mu\:}^{\left(k\right)}\left(t\right)+\sum\:_{p=1}^{\infty\:}{\xi\:}_{idp}^{\left(k\right)}{\varphi\:}_{p}^{\left(k\right)}\left(t\right)$$

where $\:{\mu\:}^{\left(k\right)}\left(t\right)$ is the mean function of the $\:k$th cluster and $\:{\xi\:}_{idp}^{\left(k\right)}$ is the $\:p$th functional principal component (FPC) score of the $\:i$th individual on the $\:d$th day within the $\:k$th cluster, associated with the eigenfunction $\:{\varphi\:}_{p}^{\left(k\right)}\left(t\right)$ for all $\:p\ge\:1$. We truncate the FPCs to a finite vector for dimension reduction, which results in the information in $\:{A}_{id}^{\left(k\right)}\left(t\right)$ being represented by a $\:P$-dimensional vector$\:\:{{\Xi\:}}_{id}^{\left(k\right)}=\left({\xi\:}_{id1}^{\left(k\right)},\:\dots\:.,{\xi\:}_{idP}^{\left(k\right)}\right)$, where $\:P$ is the number of retained principal components. Then, the mean activity function $\:{A}_{id}^{\left(k\right)}\left(t\right)$ can be expressed in the following form:

11 $$\:{A}_{id}^{\left(k\right)}\left(t\right)={\mu\:}^{\left(k\right)}\left(t\right)+\sum\:_{p=1}^{P}{\xi\:}_{idp}^{\left(k\right)}{\varphi\:}_{p}^{\left(k\right)}\left(t\right)$$

We then investigate whether each person’s FPC score on each day, representing the subject-specific pattern in each cluster, can serve as a critical digital biomarker to explore the relationship between PA and health-related outcomes.

### Applying digital biomarkers in association studies or classification problems

#### Application in association studies

Given the substantial daily variation observed among individuals, each day’s data is treated independently in model building. The association model can be expressed as follows:

12 $$\:g\left(E{(Y}_{i})\right)={\beta\:}_{0}+\sum\:_{k=1}^{K}\sum\:_{p=1}^{P}{\beta\:}_{p}^{\left(k\right)}{\xi\:}_{idp}^{\left(k\right)}+{\varvec{Z}}_{i}{\varvec{\alpha\:}}^{T}$$

where $\:{Y}_{i}$ is the health outcome for the $\:i$th individual, $\:{\xi\:}_{idp}^{\left(k\right)}$ is the $\:i$th individual’s $\:p$th FPC score on the $\:d$th day within the $\:k\text{t}\text{h}$ cluster, $\:{\varvec{Z}}_{i}$ is the $\:i$th individual’s covariates such as gender, age, etc., and $\:g\left(\bullet\:\right)$ is a link function. If one considers the correlation between data collected on different days for an individual and treats it as repeated measures data, a generalized linear mixed effects model can be employed for analysis.

#### Application in classification problems

Another application of these cluster-based digital biomarkers is classification with machine learning methods. Machine learning models can be developed by using the defined digital biomarkers, $\:{\xi\:}_{idp}^{\left(k\right)}$, and covariates, $\:{\varvec{Z}}_{i}$, as features to perform classification tasks and make predictions about health outcomes.

### Three application studies

#### NHANES surveys in 2011–2012

The first study utilized data from the 2011–2012 National Health and Nutrition Examination Survey (NHANES), which is representative of the civilian, non-institutionalized population of the United States [40]. PA was measured using the ActiGraph GT3X + device, capable of recording acceleration at an 80 Hz sampling rate. The raw data were aggregated to the minute level by calculating the median of all points within each minute. Each participant had up to 194 h of accelerometer data collected.

A total of 552 children aged 6 to 12 years with triaxial accelerometer data were selected for analysis, including 276 children with normal weight and 276 with obesity. For the preprocessing of PA data, only days with complete 24-hour data were considered. After preprocessing, 3,827 days of data were analyzed, with 1,914 days from children with obesity and 1,913 days from normal-weight children.

#### Depresjon Study

The second study, the Depresjon study, assessed participants mental health using the Montgomery-Asberg Depression Rating Scale (MADRS) [41]. Of the 55 participants, 23 exhibited severe depression (MADRS score > 30), while 32 served as a control group without depressive symptoms. The activity count value was derived from raw acceleration per minute data recorded by the Actiwatch at 32 Hz. Rigorous data quality control eliminated invalid records. As a result of the data preprocessing, 739 days were included in the analysis, consisting of 306 days from depressed patients with unipolar or bipolar and 433 days from controls. Each day contained 1,440 activity counts, each representing a one-minute total activity count ranging from 0 to 3,000. The collected activity counts were divided by 1,000 to ensure manageable amplitude values.

#### PSYKOSE Study

The third study, the PSYKOSE study, involved 22 participants with schizophrenia and a control group of 32 healthy participants [42]. Similar to the Depresjon study, the Actiwatch was used to record daily activity, and the same data pre-processing was applied to ensure data quality. After data preprocessing, 729 days were included in the analysis, consisting of 296 days for patients with schizophrenia and 433 days for controls.

#### Logistic Regression for Association Investigation

The association model for the Depresjon and PSYKOSE studies can be expressed as follows

13 $$\:logit\left(E\left({Y}_{i}\right)\right)={\beta\:}_{0}+\sum\:_{k=1}^{K}\sum\:_{p=1}^{P}{\beta\:}_{p}^{\left(k\right)}{\xi\:}_{idp}^{\left(k\right)}+{Gender}_{i}+Ag{e}_{i}$$

where $\:{Y}_{i}=1$ if the $\:i$th individual is a patient (diagnosed with depression in Depresjon study and schizophrenia in the PSYKOSE study) and 0 otherwise, $\:{Gender}_{i}=1$ for male and 0 for female, $\:Ag{e}_{i}=1$ if the individual is aged between 40 and 69.

#### Competing classification models

Five classifiers (Naive Bayes, SVM, logistic regression with lasso penalty, decision tree, and random forest) were constructed, and the performance was evaluated by accuracy, sensitivity, and specificity. To account for the inter-individual variability and uncertain disease onset, a leave-one-subject-out cross-validation combined with majority voting was employed. Each subject was taken in turn as the testing data while the remaining subjects formed the training data set. Since each individual had data for multiple days, daily predictions were made for each subject, and majority voting across these daily predictions determined the final decision. For instance, if a person has five days of data, the classification model will generate five predictions. If the person is predicted to be a case for three out of five days, the final prediction will be that the person is a case. The feature set for all classifiers was identical to that used in the association model.

## Results

### Application 1: The NHANES study

#### Constructing and investigating Digital biomarkers

Our proposed elastic distance-based motif clustering method was applied to discover activity patterns and to construct digital biomarkers for three real-world applications. We evaluated the impact of different time window sizes (15 and 30 min). For the NHANES study, a 15-minute window is optimal for analyzing children’s activity patterns. This may be due to children’s shorter attention spans and engagement in more frequent short-duration activities. When examining longer time windows, we observed that many motifs could be composed of several 15-minute motifs (Supplementary Figure S5, Figure S6, Figure S7). Consequently, our primary analysis emphasizes a 15-minute window, with results from other window sizes detailed in the supplementary material.

**Fig. 1:**
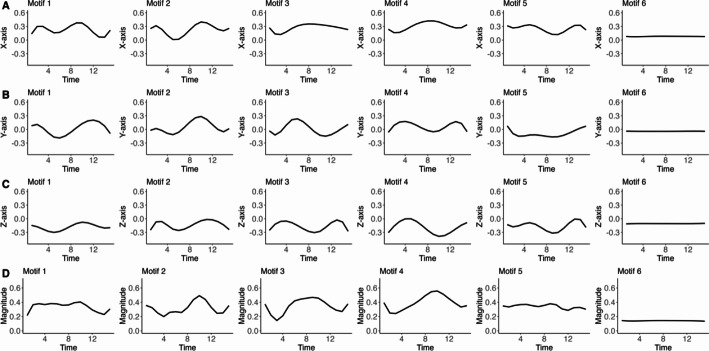
Visualization of the mean activity function ($\:\frac{1}{n}\sum\:_{i=1}^{n}\frac{1}{D}\sum\:_{d}{A}_{id}^{\left(k\right)}\left(t\right)$) for each cluster representing different motifs identified by the elastic distance-based motif clustering algorithm in the NHANES study. The mean activity function of the triaxial accelerometer is presented in (**A**) the X-axis, (**B**) the Y-axis, (**C**) the Z-axis, and (**D**) the combined magnitude of activity, calculated as $\:\sqrt{{x\left(t\right)}^{2}+{y\left(t\right)}^{2}+z{\left(t\right)}^{2}}$

The elastic distance-based motif clustering method identified distinct activity patterns (motifs) from the triaxial accelerometer data, resulting in six motifs for each axis. Subsequently, 18 digital biomarkers were derived from the three axes. Figure 1 illustrates the clustering outcomes, displaying the mean activity function for each aligned three-axis function. It highlights the differences in activity patterns across the three axes, demonstrating the effectiveness of the elastic distance-based motif clustering algorithm in handling multi-axis data and uncovering meaningful patterns.

**Fig. 2:**
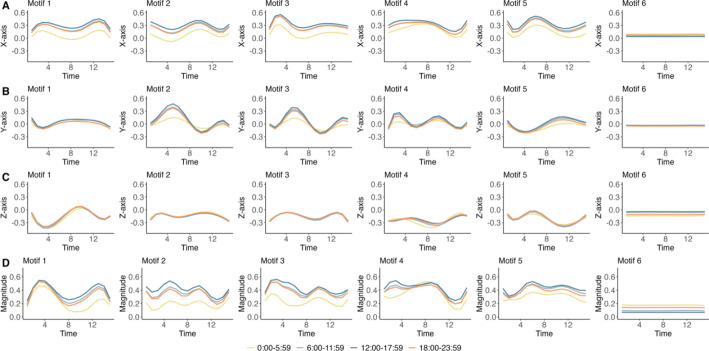
Visualization of mean activity functions across four time intervals representing different motifs obtained by the elastic distance-based motif clustering algorithm in NHANES. The mean activity functions of the triaxial accelerometer are presented in (**A**) the X-axis, (**B**) the Y-axis, (**C**) the Z-axis, and (**D**) the combined magnitude of activity, calculated as $\:\sqrt{{x\left(t\right)}^{2}+{y\left(t\right)}^{2}+z{\left(t\right)}^{2}}$

To explore the relationship between diverse activity patterns and children’s daily routines, we partitioned the 24-hour day into four 6-hour time intervals. We computed the frequency distribution of specific activity functions across these time intervals within each cluster for each group. The results are shown in Fig. 2 and Supplementary Table S1. Motif 6 shows a consistently low level of activity (Fig. 2), suggesting it may represent sleep or sedentary behavior. This interpretation is supported by a temporal distribution wherein approximately 45% of the associated curves are localized within the nocturnal period of 00:00 and 05:59. The frequency of Motif 4 (in Fig. 2) remained relatively consistent across all four time intervals, suggesting it may represent a time-independent activity pattern with limited behavioral specificity.

### Application 2 and 3: Two mental health studies

#### Constructing and investigating digital biomarkers

For the Depresjon and PSYKOSE datasets, a 30-minute window is more suitable for capturing patients’ activity patterns. Consequently, our primary analysis emphasizes these specific time windows, with results from other window sizes detailed in the supplementary material. In addition, we determined the optimal number of clusters ($\:k$) based on the within-cluster variation to between-cluster variation ratio, Silhouette width, and the classification performance of the resulting digital biomarkers. The elastic distance-based motif clustering algorithm assigned cluster labels to activity functions within the entire dataset, resulting in the derived digital biomarkers.

**Fig. 3:**
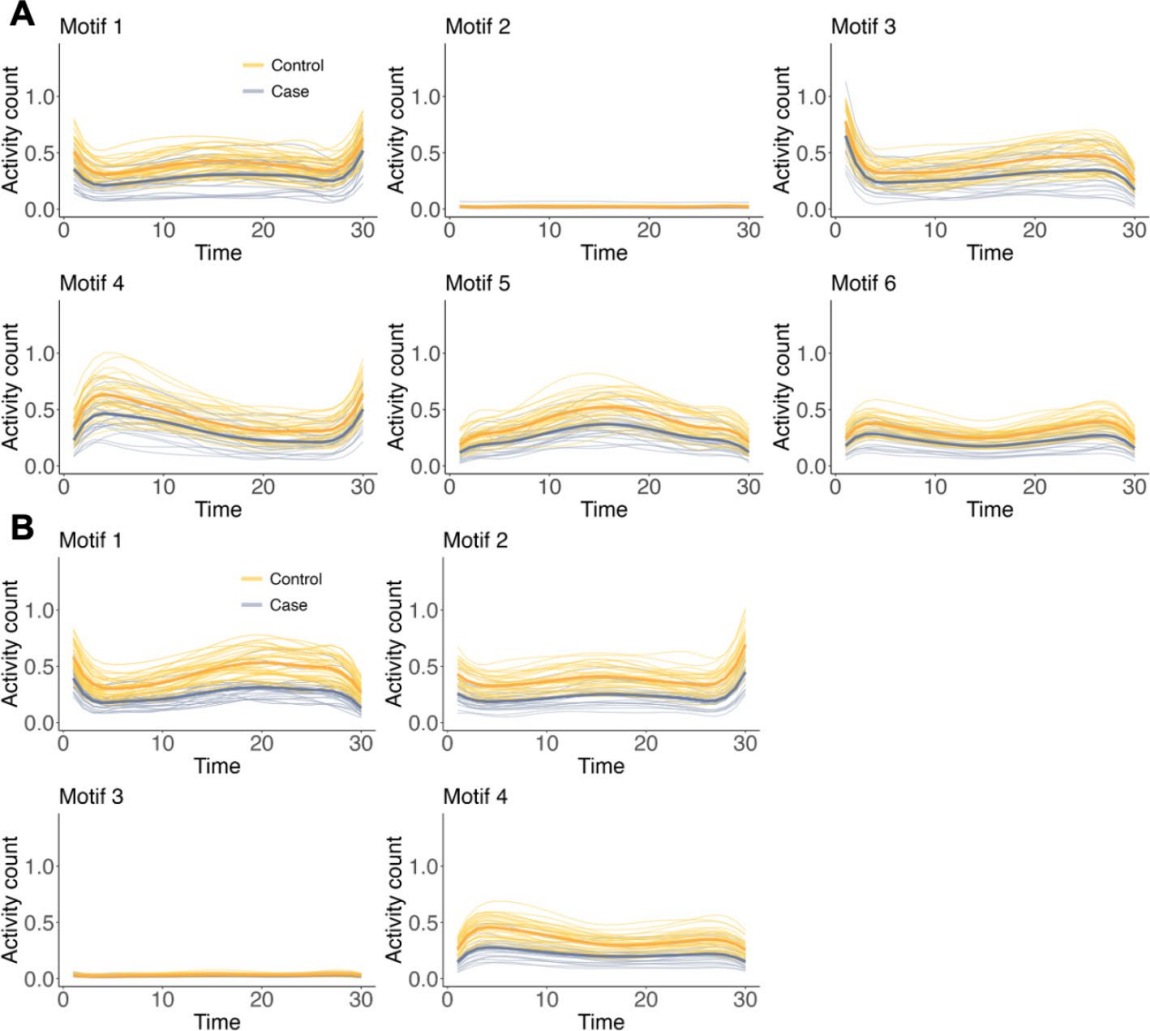
Visualization of mean activity functions ($\:\frac{1}{D}\sum\:_{d}{A}_{id}^{\left(k\right)}\left(t\right)$) for each individual representing different motifs obtained by the elastic distance-based motif clustering algorithm in (**A**) Depresjon study and (**B**) PSYKOSE study

The Depresjon study yielded six motifs (clusters) and 12 digital biomarkers, while the PSYKOSE study identified four motifs and eight digital biomarkers. Figure 3 illustrates the clustering results, presenting the mean activity function for each individual within their respective clusters. While activity patterns within each cluster are similar, distinct patterns emerge across different clusters. The activity count patterns vary among motifs. Some motifs (Motifs 2 in Fig. 3A; Motifs 3 in Fig. 3B) exhibit relatively stable activity counts, whereas other motifs (Motifs 1, 3, 4, and 5 in Fig. 3A; Motifs 1 and 2 in Fig. 3B) demonstrate significant fluctuations. On average, the activity counts of the control group were higher than those of the case group, suggesting that the control group had a more active lifestyle during the study period.

**Fig. 4:**
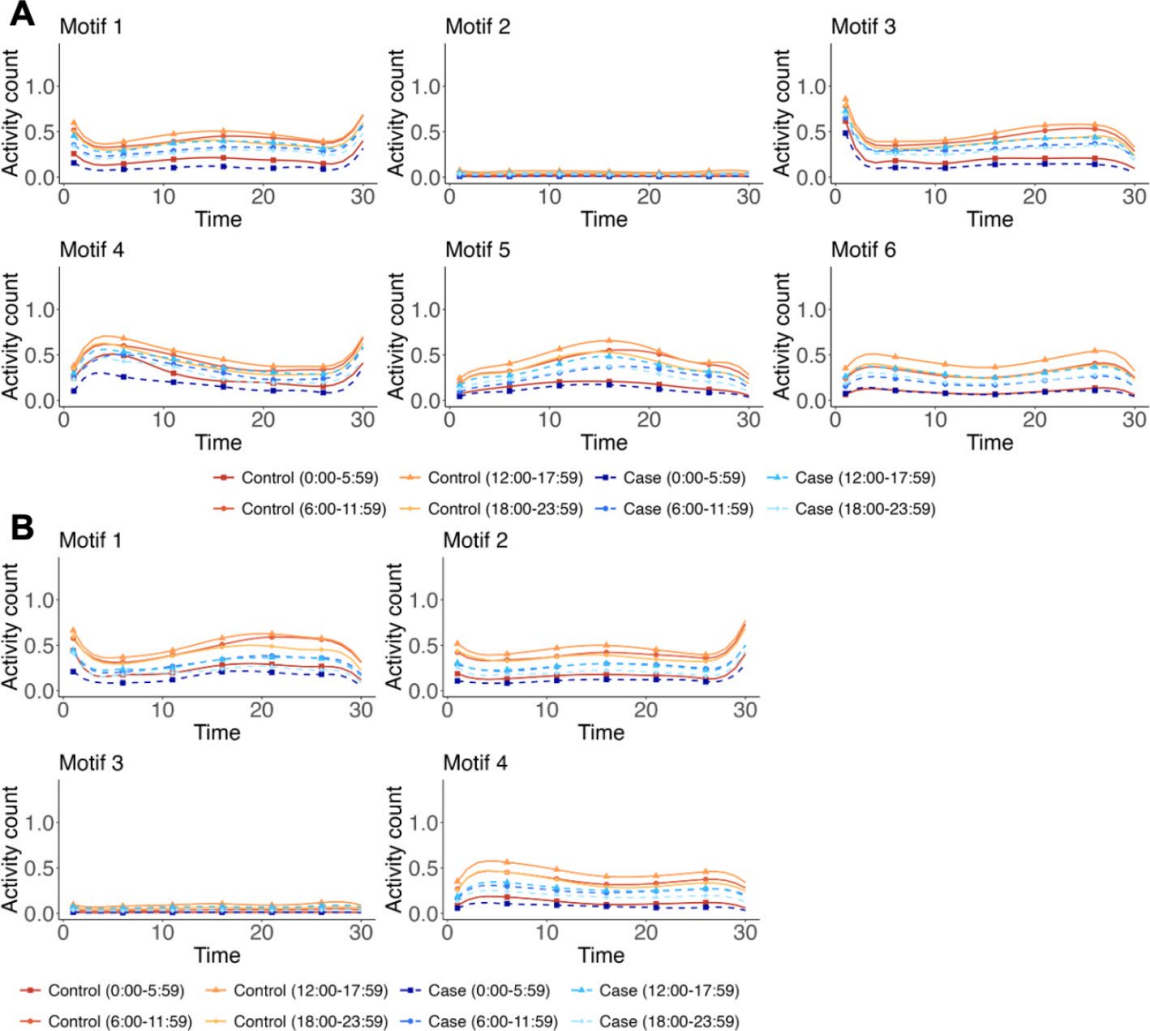
Visualization of mean activity functions for different groups across the four time intervals representing different motifs obtained by the elastic distance-based motif clustering algorithm in (**A**) Depresjon study and (**B**) PSYKOSE study

We explored the relationship between diverse activity patterns and individuals’ daily routines. We partitioned the 24-hour day into four time intervals and computed the frequency distribution of specific activity functions across these time intervals within each cluster for each group. This allows us to identify the activity patterns most commonly observed in particular time intervals for each group. The results are shown in Fig. 4, Table S2, and Table S6. The low-activity motifs, such as Motif 2 (Fig. 4A) and Motif 3 (Fig. 4B), exhibit a temporal distribution strongly associated with sleep, with nearly half of the associated curves occurring between midnight and 5:59 AM. In contrast to activity patterns with lower activity counts, those with higher activity counts are predominantly observed within diurnal periods and the pre-sleep phase (over 85%). Additionally, we examined the associations between demographic variables (age and gender) and various activity patterns. No significant differences were observed across these motifs. Detailed results are presented in Supplementary Figure S9, Figure S10, Table S2, and Table S6.

We further examined disease-specific patterns within each motif. Controls exhibited higher overall activity levels (Fig. 3), particularly among younger individuals (Supplementary Figure S9, Table S5, and Table S9). This discrepancy might be attributed to the relatively homogeneous nature of the hospitalized case group. Furthermore, while females in the control group exhibited higher activity levels than males, no significant gender differences were observed in the case group of the Depresjon study. Conversely, females in the case group of the PSYKOSE study displayed lower activity levels (Supplementary Figure S10, Table S4, and Table S8). When examining temporal distribution (Fig. 4, Supplementary Table S3, and Table S7), healthy individuals exhibited more regular diurnal rhythms. Low-activity motifs (Motif 2 for Depresjon and Motif 3 for PSYKOSE) predominantly occurred between midnight and 5:59 AM, and motifs with higher activity counts were more frequent during diurnal periods and the pre-sleep phase. In contrast, patients showed disrupted circadian rhythms, with increased nighttime and reduced daytime activity. This was particularly evident in the depression group.

#### Association studies for the two mental health studies

Functional Principal Component Analysis was applied to each motif to reduce dimensionality and extract meaningful features, resulting in digital biomarkers. Since the top two FPCs in both studies explained approximately 80% of the variation across all motifs, these were selected as representative features. Their corresponding FPC scores were then used to construct regression models. As shown in Supplementary Figures S11A and S12A, the first eigenfunctions of all motifs exhibit variations in activity intensity. Moreover, the second eigenfunctions demonstrate phase shift.

To identify influential activity patterns between the two groups, logistic regression with lasso penalty was employed to select significant digital biomarkers. The results are presented in Table 1. In the Depresjon study, FPC 1 in Motif 1, 5, and 6 exhibited a significant effect between the two groups; that is, these three activity patterns were associated with depression. In the PSYKOSE study, gender was excluded because the observations were perfectly separated by gender, and the logistic regression estimation algorithm did not converge. The model for the PSYKOSE study was established without considering gender. FPC 1 in Motif 1, 2, and 4 and FPC 2 in Motif 4 demonstrated a significant effect between the two groups; that is, these three activity patterns were associated with schizophrenia.

**Table 1:** Multiple regression models for mental health (effect estimates and 95% confidence intervals) in the two application studies

Variable	Odd ratio	*P*-value
**Depresjon study**		
Motif 1 FPC 1	0.68 (0.54, 0.86)	0.002
Motif 5 FPC 1	0.68 (0.54, 0.86)	0.001
Motif 6 FPC 1	0.5 (0.35, 0.72)	< 0.001
Age (40–69)	1.72 (1.23, 2.40)	0.001
Gender (Male)	1.76 (1.27, 2.46)	0.001
**PSYKOSE study**		
Motif 1 FPC 1	0.35 (0.24, 0.51)	< 0.001
Motif 2 FPC 1	0.46 (0.31, 0.69)	< 0.001
Motif 4 FPC 1	0.47 (0.29, 0.77)	0.002
Motif 4 FPC 2	12.53 (3.58, 43.85)	< 0.001
Age (40–69)	1.97 (1.35, 2.9)	0.001

#### Classification models for the two mental health studies

Table 2 presents the performance of five classification models. To establish a baseline for comparison, a classifier was created using only demographic information (age and gender) to assess the model’s basic ability to distinguish between case and control groups. The classification model incorporating digital biomarkers consistently outperformed the baseline model in the two studies. Incorporating digital biomarkers increased sensitivity in both studies, with a 0.35 increase in the Depresjon study and a 0.32 increase in the PSYKOSE study for naïve Bayes. This indicates that digital biomarkers are effective in discriminating the case group. Moreover, in the PSYKOSE study, the digital biomarkers improved specificity, aiding in the detection of the control group; however, their impact on identifying healthy individuals in the Depresjon study was less pronounced.

**Table 2:** Performance of classification model for mental health in the two application studies

	Accuracy			Sensitivity		Specificity
**Depresjon study**						
**Baseline model** (age, gender)						
Naïve Bayes	0.64			0.35		0.84
SVM	0.64			0.35		0.84
Logistic regression (Lasso)	0.64			0.35		0.84
Decision Tree	0.64			0.35		0.84
Random forests	0.62			0.30		0.84
**12 digital biomarkers (6 motifs, 2 FPCs per motif) + no demographics**						
Naïve Bayes	0.67			0.70		0.66
SVM	0.67			0.39		0.88
Logistic regression (Lasso)	0.67			0.52		0.78
Decision Tree	0.62			0.26		0.88
Random forests	0.71			0.43		0.91
**12 digital biomarkers (6 motifs, 2 FPC per motif) + demographics**						
Naïve Bayes	0.62			0.65		0.59
SVM	0.73			0.48		0.91
Logistic regression (Lasso)	0.62			0.48		0.72
Decision Tree	0.62			0.26		0.88
Random forests	0.75			0.52		0.91
**PSYKOSE** **study**						
**Baseline model** (age, gender)						
Naïve Bayes		0.72	0.59		0.81	
SVM		0.61	0.59		0.62	
Logistic regression (Lasso)		0.61	0.59		0.62	
Decision Tree		0.61	0.59		0.62	
Random forests		0.65	0.59		0.69	
**8 digital biomarkers (4 motifs, 2 FPCs per motif) + no demographics**						
Naïve Bayes		0.83	0.91		0.78	
SVM		0.85	0.77		0.91	
Logistic regression (Lasso)		0.83	0.82		0.84	
Decision Tree		0.83	0.64		0.97	
Random forests		0.87	0.82		0.91	
**8 digital biomarkers (4 motifs, 2 FPCs per motif) + demographics**						
Naïve Bayes		0.85	0.91		0.81	
SVM		0.81	0.68		0.91	
Logistic regression (Lasso)		0.83	0.77		0.88	
Decision Tree		0.80	0.64		0.91	
Random forests		0.85	0.73		0.94	

## Discussion

This study proposed an elastic distance-based motif clustering algorithm for identifying motifs in free-living PA data and then utilizing the FPCA to define digital biomarkers from each motif. The results of this research underscore the effectiveness of grouping similar activity patterns, thereby facilitating the acquisition of digital biomarkers for subsequent analysis. The proposed method is not only effective but also offers a novel perspective for managing free-living data, instilling confidence in its potential.

The results of the NHANES study indicated the feasibility of using the clustering algorithm for three-axis data. In Depresjon and PSYKOSE study, each motif suggests that individuals with mental health issues exhibit lower levels of daily PA, corroborating findings from previous studies. The clustering approach provides a more meaningful understanding of the data, revealing that patients who exhibit specific activity patterns with more intensity tend to be associated with lower disease risk.

The sensitivity of k-means clustering algorithms to initial values is a well-documented challenge. To address this, our proposed method adopts a straightforward approach by randomly choosing $\:K$ activity segments from the original dataset as initial cluster centers. This simple yet effective strategy provides a flexible starting point, especially when domain-specific knowledge is limited. Nonetheless, the method allows for customization, enabling researchers to incorporate prior knowledge or other preferred choices to refine the initial cluster centers.

Prior research on triaxial accelerometer data has commonly assumed independence among the axes, as highlighted by Mizell [43]. Following this common practice, our study also independently treats data from each axis. Each axis’s data undergoes SRVF transformation, and distances are calculated separately before being simply summed to obtain a combined multi-axis distance for subsequent analysis. While it is possible to consider the correlation between multiple axes by performing SRVF transformation on the entire multi-axis data simultaneously, as detailed in Srivastava et al. [44] and Kurtek et al. [45], this approach was not adopted in our study. Future research could explore the impact of this assumption on the analysis results. Furthermore, treating each axis as independent offers the flexibility to assign weights to different axes when analyzing specific activities or states. By assigning a higher weight to the axis which is most representative of the activity, we can tailor the distance calculation to better capture the nuances of different activity patterns. This flexibility allows us to account for the varying significance of different axes in other research questions or datasets.

This study’s simplistic approach to time window segmentation is a limitation of this study. However, the potential impact of a more sophisticated method on the accuracy of the study is significant. While we experimented with a fixed-length window based on the approximate duration of daily activities, a more sophisticated method that accounts for overlapping segments could significantly enhance the accuracy of capturing the dynamics of human behavior, especially considering more complex and nuanced activity patterns.

Another limitation of this study is that the digital biomarkers defined based on the motifs identified by the proposed algorithm require further research to understand the connection between each motif and the actual activities. Given that cluster analysis is an exploratory data analysis method, further investigation is necessary to delve deeper into the meaning of each cluster after obtaining the clustering results. Similarly, the activity patterns identified from free-living activity data using the proposed algorithm need additional research to understand the types of activities they represent, enabling their integration with clinical questions and providing greater clinical significance and value in addressing clinical problems.

In summary, the motif clustering method is valuable for labeling free-living activity patterns. The clustering-based digital biomarkers, within the framework developed in this study, offer a novel perspective on the relationship between specific PA patterns and health outcomes. Beyond one-dimensional activity count functions, this framework is adaptable to a wide range of multivariate functional data, reassuring its applicability. By applying this method, the resulting digital biomarkers hold significant promise for advancing personalized health assessment and disease detection, shaping a positive future for healthcare.

## Electronic supplementary material

Below is the link to the electronic supplementary material.

Supplementary Material 1

## Data Availability

The dataset of the Depresjon study can be accessed at http://datasets.simula.no/depresjon/, the dataset of PSYKOSE at https://datasets.simula.no/psykose/ and the dataset of NHANES at https://www.cdc.gov/nchs/nhanes/index.htm.

## Abbreviations

PA: Physical activity
FDA: Functional data analysis
FPCA: Functional principal component analysis
FPC: Functional principal component
SRVF: Square root velocity function

## Code availability

The code is available from the authors upon request.
