# Correction: Motif clustering and digital biomarker extraction for free-living physical activity analysis

**DOI:** 10.1186/s13040-025-00449-6

**Published:** 2025-05-12

**Authors:** Ya-Ting Liang, Charlotte Wang

**Affiliations:** 1 Institute of Epidemiology and Preventive Medicine, College of Public Health, National Taiwan University, Taipei, Taiwan; 2 Institute of Health Data Analytics and Statistics, College of Public Health, National Taiwan University, No. 17, Xu-Zhou Road, Taipei, 100025 Taiwan; 3 Master of Public Health Program, College of Public Health, National Taiwan University, Taipei, Taiwan

**Correction** **:** **BioData Mining 18, 8 (2025)**

**https://doi.org/10.1186/s13040-025-00424-1**

Following publication of the original article [1], the authors identified two errors.

In formula 4, we used incorrect symbols to describe the calculation of phase distance. This issue stems from referencing Dr. Srivastava's work on elastic shape analysis without initially noticing that symbol usage and definitions varied across different papers in his series. We inadvertently used symbols in Equation (4) consistent with one subset, leading to a notational clash within our paper compared to the surrounding text and established conventions. We will correct the symbols in Equation (4) to resolve this inconsistency. This correction is purely related to the mathematical notation and does not affect our paper's research findings, methodology, or conclusions. The underlying calculations and results remain the same.

The incorrect formula 4 is given below:

$${d}_{phs}\left({f}_{1},{f}_{2}\right)=\text{arccos}\left(\langle {q}_{l}, {q}_{\gamma }\rangle \right)$$

The correct formula 4 is given below:

$${d}_{phs}\left({f}_{1}, {f}_{2}\right)={\text{cos}}^{-1}\left({\int }_{0}^{1}\sqrt{\dot{l\left(t\right)}}\sqrt{\dot{\gamma \left(t\right)}}dt\right)={\text{cos}}^{-1}\left({\int }_{0}^{1}\sqrt{1}\sqrt{\dot{\gamma \left(t\right)}}dt\right)={\text{cos}}^{-1}({\int }_{0}^{1}\sqrt{\dot{\gamma \left(t\right)}}dt)$$

In the Motif clustering algorithm, step 3, the mathematical formular, ${d}_{phs}({x}_{ij}\left(t\right), {c}^{\left(k\right)}(t))$, is based on the Equation (4), thus it needs to be corrected along with it.

The incorrect algorithm is given below:

$${d}_{phs}({x}_{ij}\left(t\right), {c}^{\left(k\right)}(t))=\text{arccos}\;(\langle {q}_{{c}^{\left(k\right)}(t)\circ l}, {q}_{{x}_{ij}(t)\circ \gamma })\rangle )$$

The correct algorithm is given below:

$${d}_{phs}\left({x}_{\mathit{ij}}\left(t\right), {c}^{\left(k\right)}(t)\right)={\mathit{cos}}^{-1}({\int }_{0}^{1}\sqrt{\dot{\gamma \left(t\right)}}dt)$$

The original article [1] has been corrected.

## References

[1] Liang YT, Wang C. Motif clustering and digital biomarker extraction for free-living physical activity analysis. BioData Mining. 2025;18:8. 10.1186/s13040-025-00424-1. 39844206 10.1186/s13040-025-00424-1 PMC11753168

