# Supplementary material for: Motif clustering and digital biomarker extraction for free-living physical activity analysis
Source: BioData Min. 2025 Jan 22;18:8. doi: 10.1186/s13040-025-00424-1 (PMC11753168; doi:10.1186/s13040-025-00424-1)
Supplement: Supplementary file 1 — Supplementary Material 1 [file 13040_2025_424_MOESM1_ESM.docx]

**Motif Clustering and Digital Biomarker Extraction for Free-Living Physical Activity Analysis**

**Supplementary**

**Application 1: NHANES study**

1. **Investigating Digital Biomarkers**


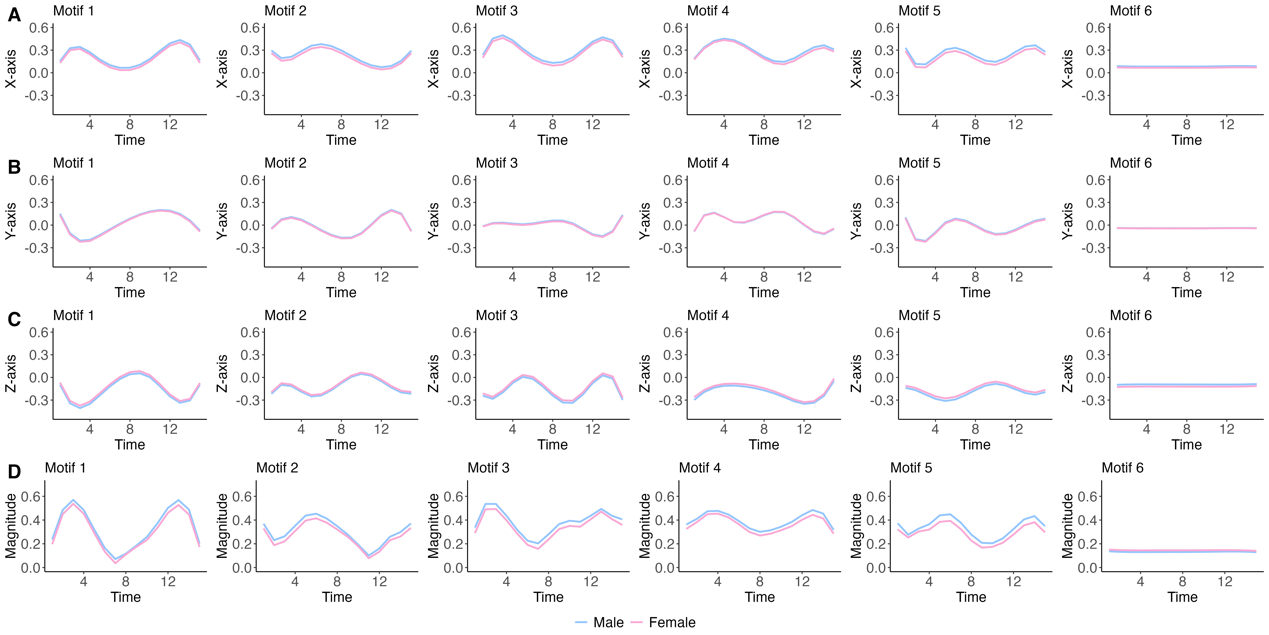


Figure S1. Visualization of the mean activity functions across different gender groups, representing distinct motifs identified using the elastic distance-based motif clustering algorithm applied to the NHANES study. The mean activity functions of the triaxial accelerometer are presented in (A) the X-axis, (B) the Y-axis, (C) the Z-axis, and (D) the combined magnitude of activity, calculated as $\sqrt{{x(t)}^{2}+{y(t)}^{2}+z\left( t \right)^{2}}$.

Table S1. Baseline characteristics of each motif in the NHANES study.

|  | Motif 1  (n = 33824) | Motif 2  (n= 82532) | Motif 3  (n = 50227) | Motif 4  (n = 17106) | Motif 5  (n = 122402) | Motif 6  (n = 61301) |
| --- | --- | --- | --- | --- | --- | --- |
| Time interval |  |  |  |  |  |  |
| 0:00-5:59 | 7142 (21.12) | 17414 (21.1) | 9253 (18.42) | 4244 (24.81) | 25693 (20.99) | 28102 (45.84) |
| 6:00-11:59 | 8903 (26.32) | 21538 (26.1) | 13733 (27.34) | 4525 (26.45) | 32166 (26.28) | 10983 (17.92) |
| 12:00-17:59 | 9259 (27.37) | 22908 (27.76) | 14641 (29.15) | 4278 (25.01) | 33975 (27.76) | 6787 (11.07) |
| 18:00-23:59 | 8520 (25.19) | 20672 (25.05) | 12600 (25.09) | 4059 (23.73) | 30568 (24.97) | 15429 (25.17) |


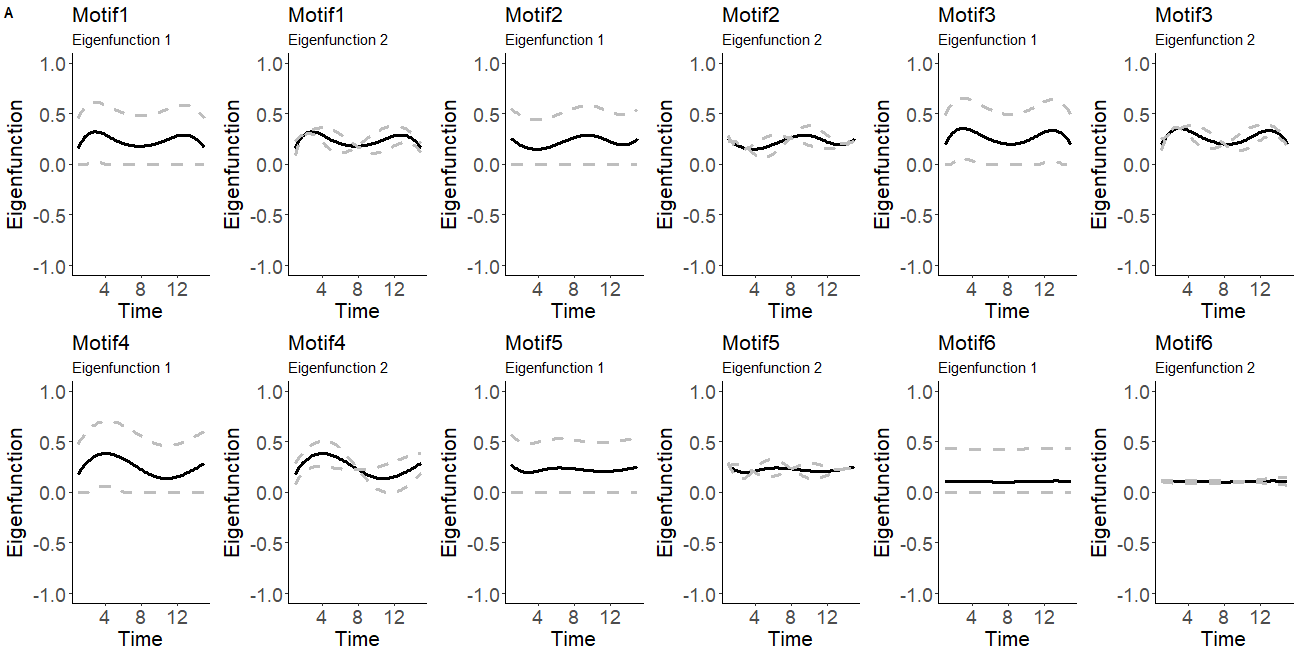


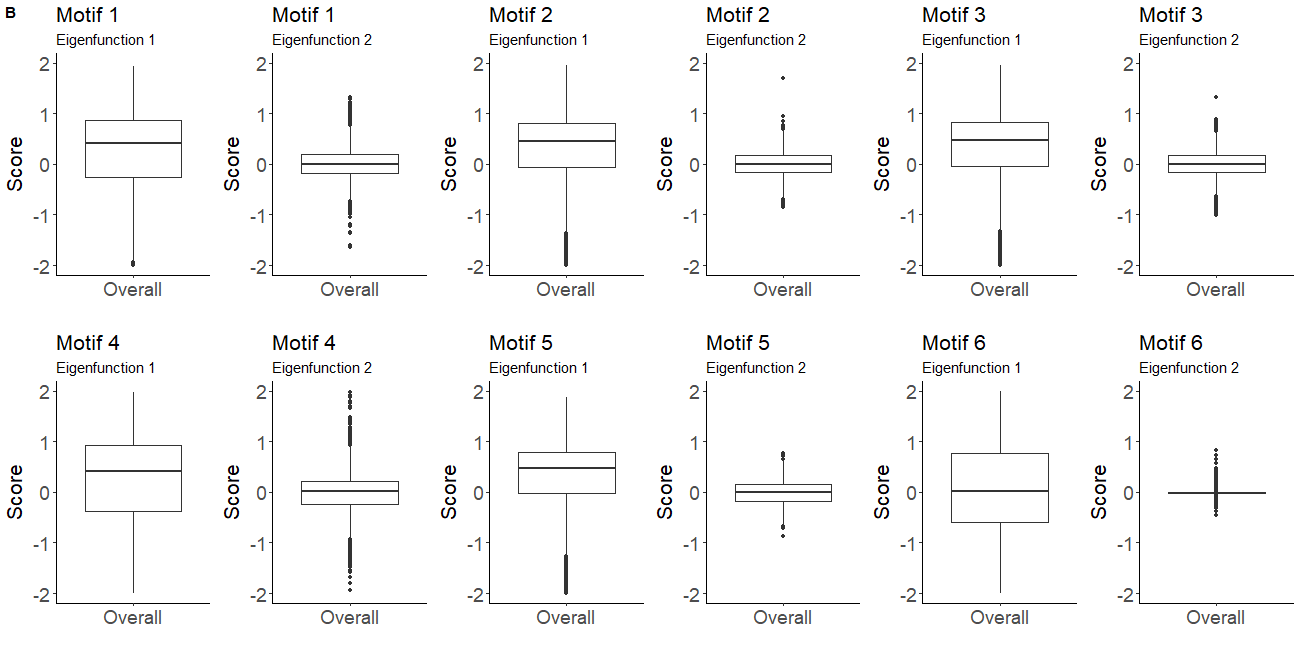


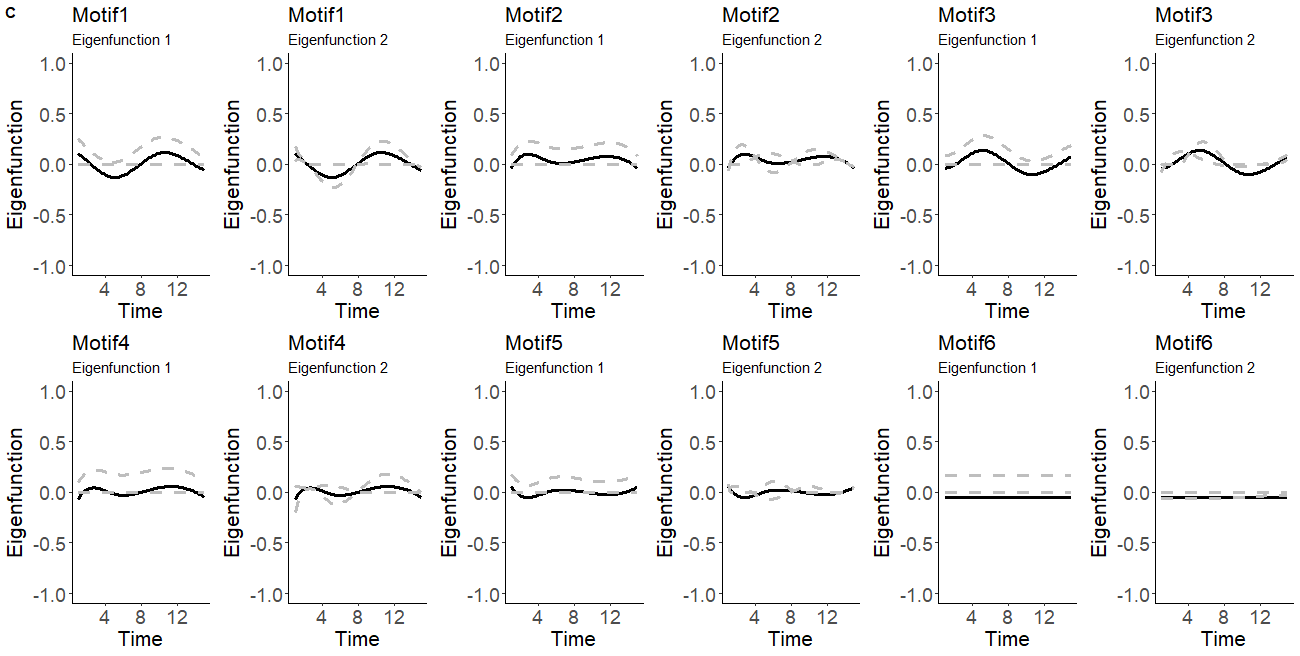


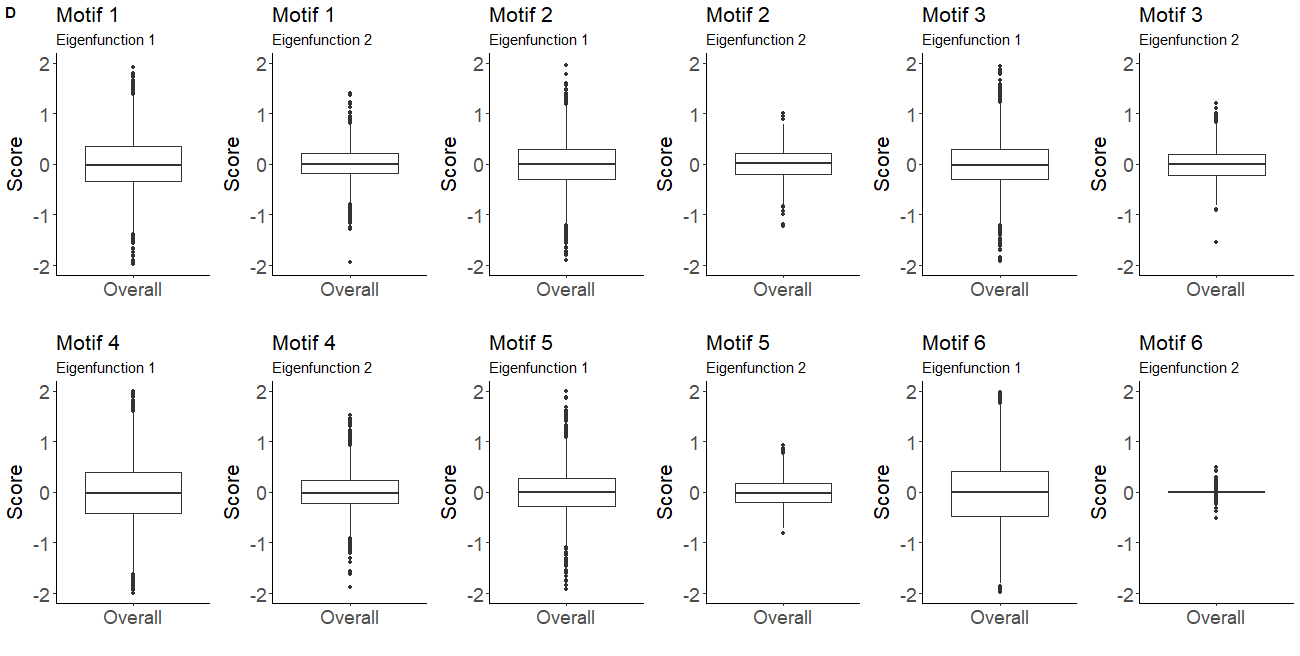


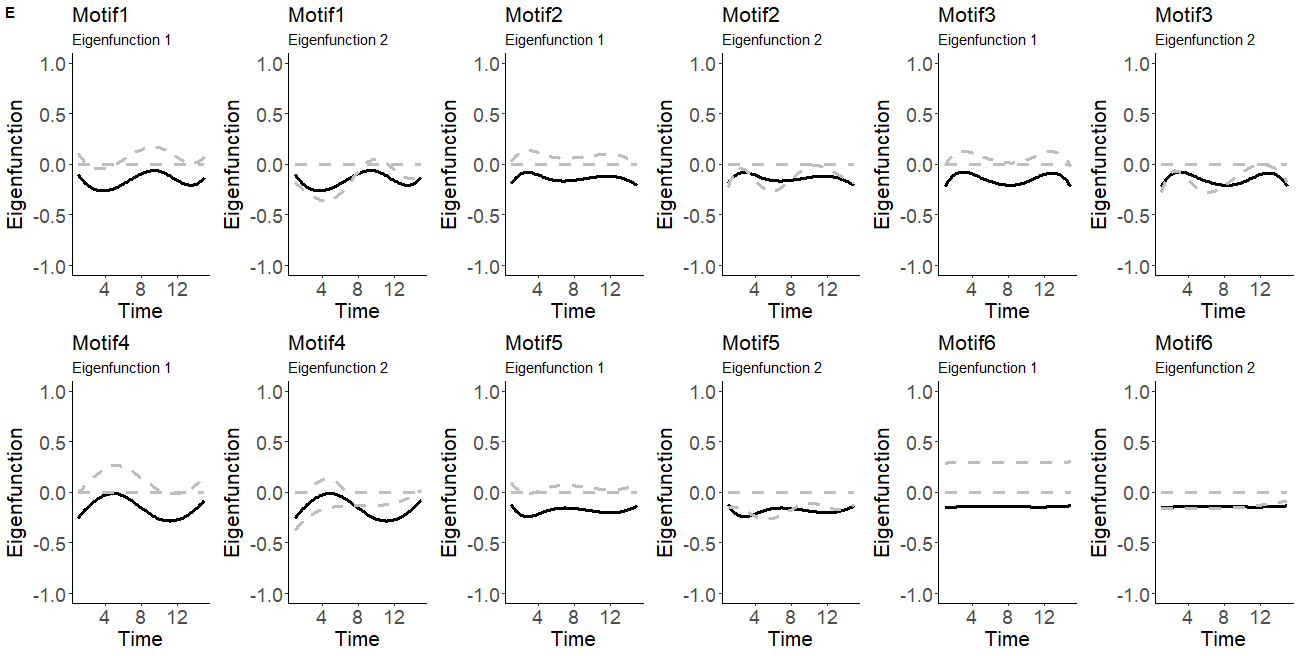


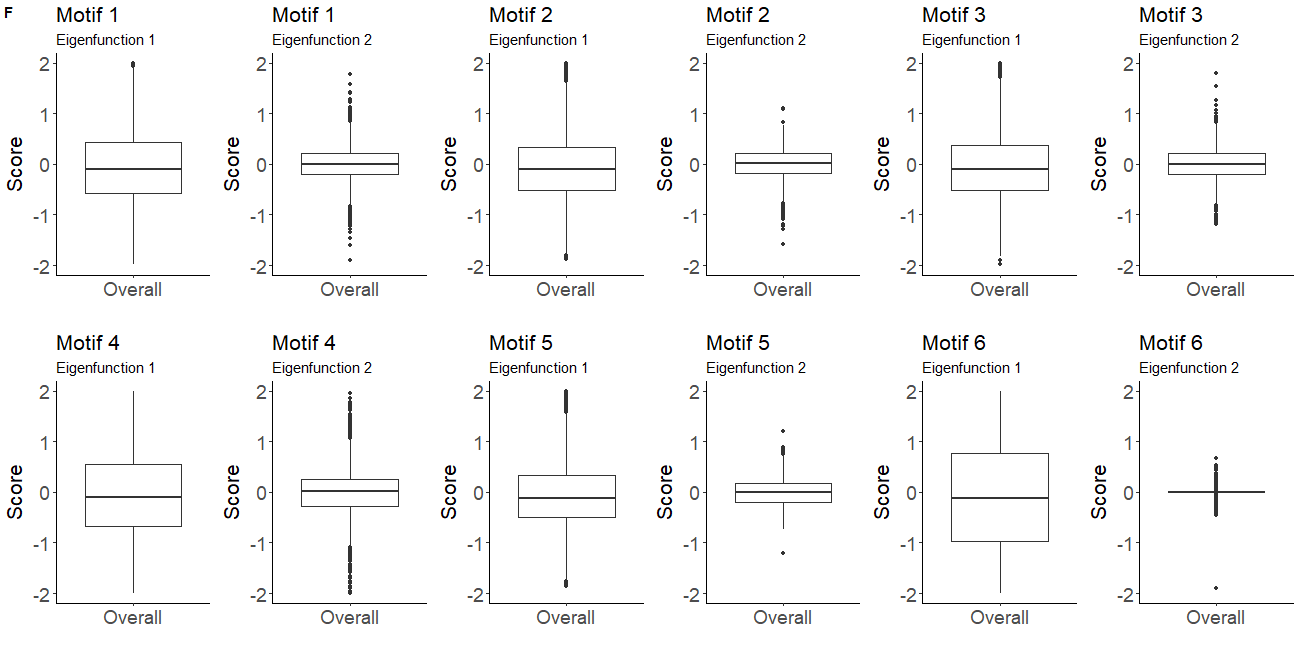


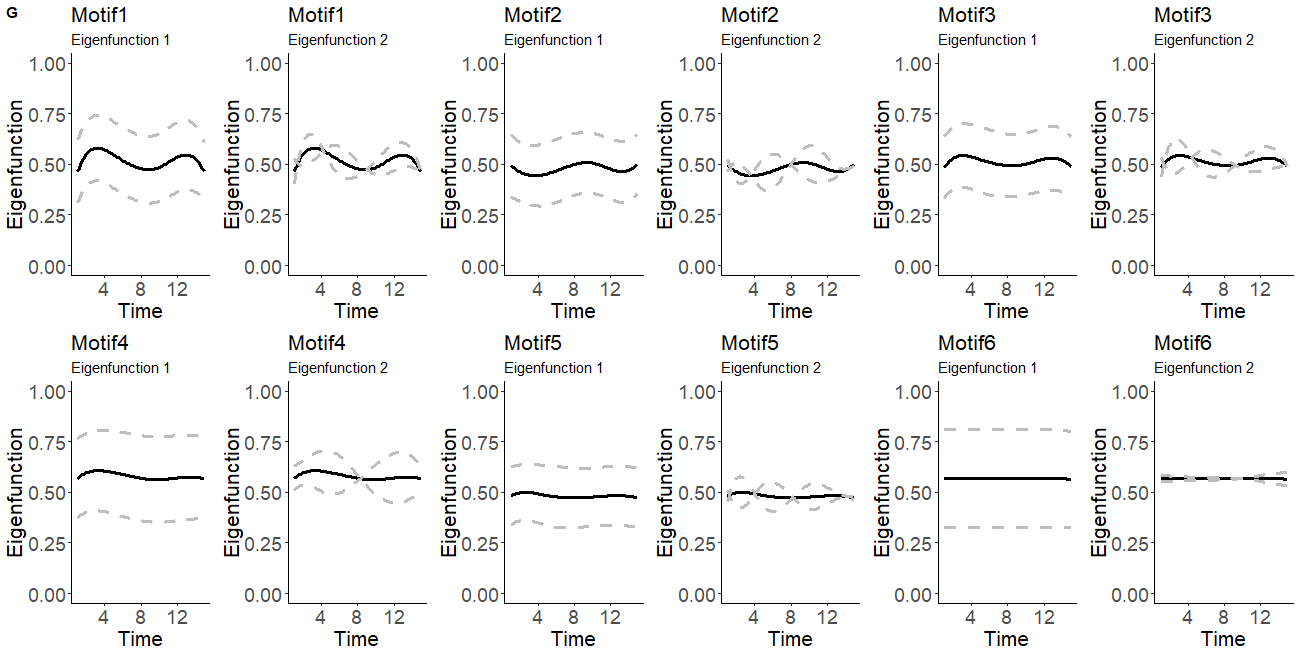


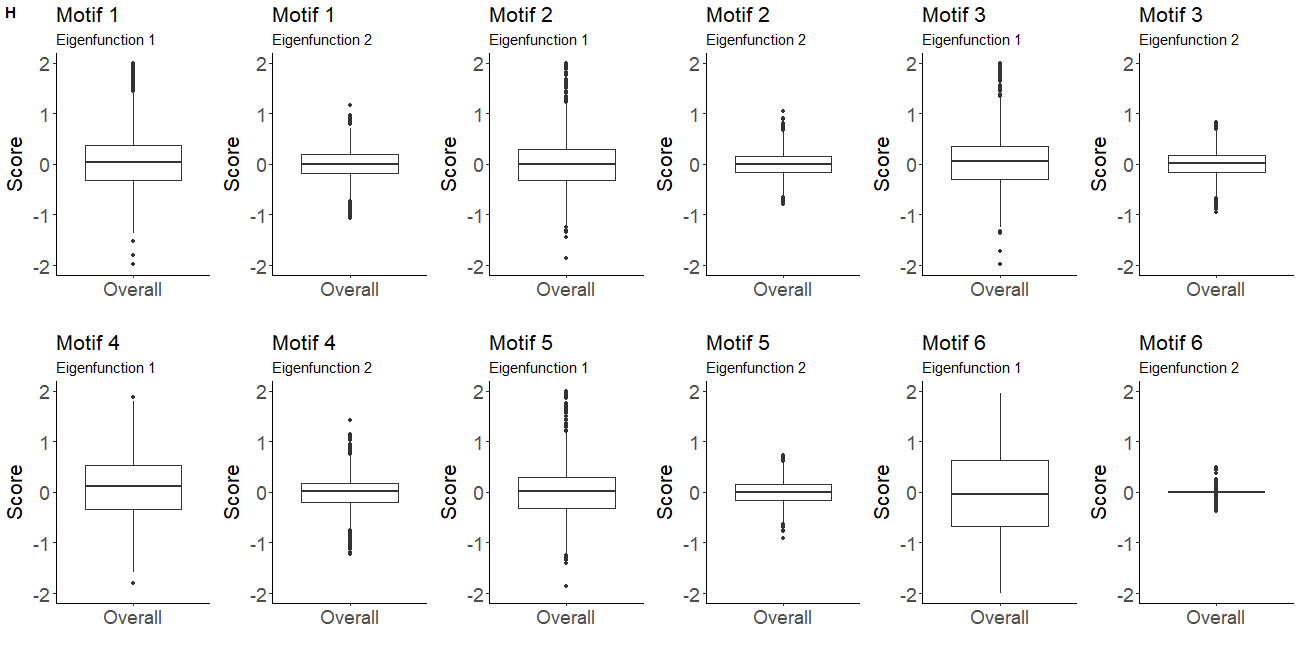


Figure S2. Visualization of the eigenfunctions and scores corresponding to FPC1 and FPC2 for various motifs derived from the NHANES study. (A) eigenfunctions for X axis; (B) FPC score for X axis; (C) eigenfunctions for Y axis; (D) FPC score for Y axis; (E) eigenfunctions for Y axis; (F) FPC score for X axis; (G) eigenfunctions for activity count (H) FPC score for activity count

1. **Activity patterns identified by the elastic distance-based motif clustering algorithm with different** $\boldsymbol{k}$ **and time windows**


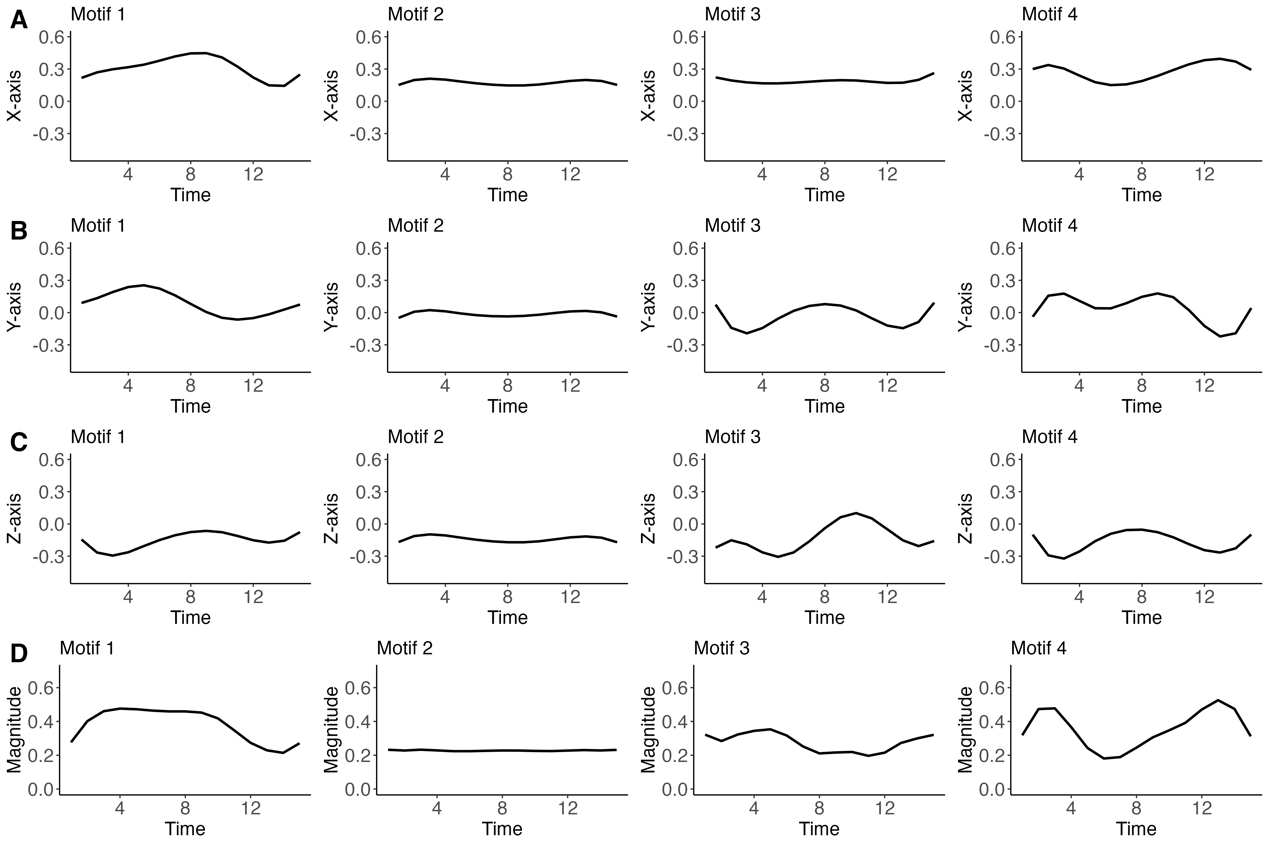


Figure S3. Visualization of the mean activity function ($\frac{1}{n}\sum_{i=1}^{n} \frac{1}{D}\sum_{d} A_{id}^{\left( k \right)}\left( t \right)$) for the four clusters representing different motifs identified by the elastic distance-based motif clustering algorithm in the NHANES study. The mean activity function of the triaxial accelerometer is presented in (A) the X-axis, (B) the Y-axis, (C) the Z-axis, and (D) the combined magnitude of activity, calculated as $\sqrt{{x(t)}^{2}+{y(t)}^{2}+z\left( t \right)^{2}}$


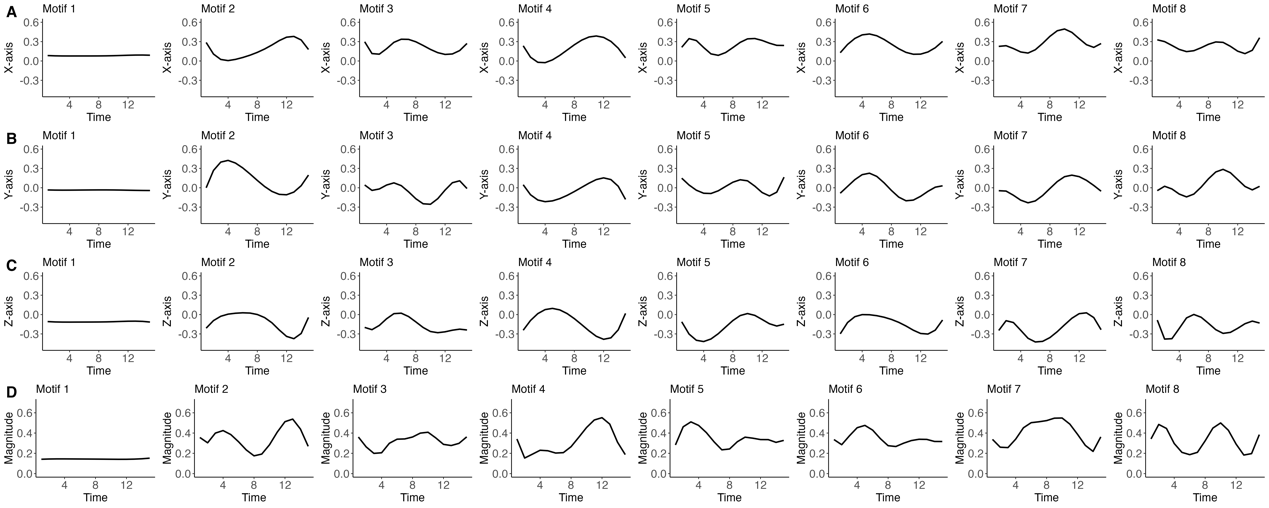


Figure S4. Visualization of the mean activity function ($\frac{1}{n}\sum_{i=1}^{n} \frac{1}{D}\sum_{d} A_{id}^{\left( k \right)}\left( t \right)$) for the eight clusters representing different motifs identified by the elastic-based motif clustering algorithm in the NHANES study. The mean activity function of the triaxial accelerometer is presented in (A) the X-axis, (B) the Y-axis, (C) the Z-axis, and (D) the combined magnitude of activity, calculated as $\sqrt{{x(t)}^{2}+{y(t)}^{2}+z\left( t \right)^{2}}$


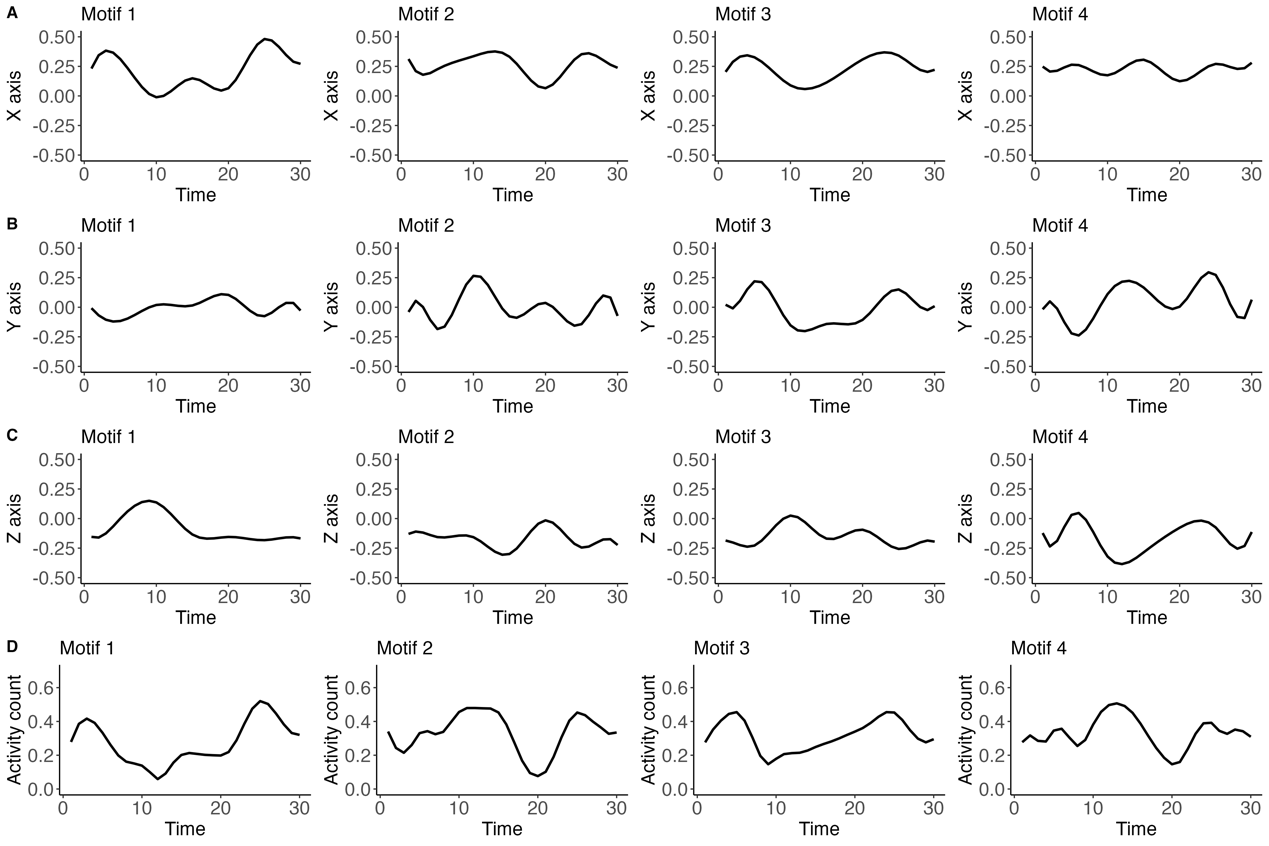


Figure S5. Visualization of mean activity functions (30 minutes) for each individual representing different motifs (4 motifs) obtained by the elastic distance-based motif clustering algorithm in the NHANES study. The mean activity function of the triaxial accelerometer is presented in (A) the X-axis, (B) the Y-axis, (C) the Z-axis, and (D) the combined magnitude of activity, calculated as $\sqrt{{x(t)}^{2}+{y(t)}^{2}+z\left( t \right)^{2}}$


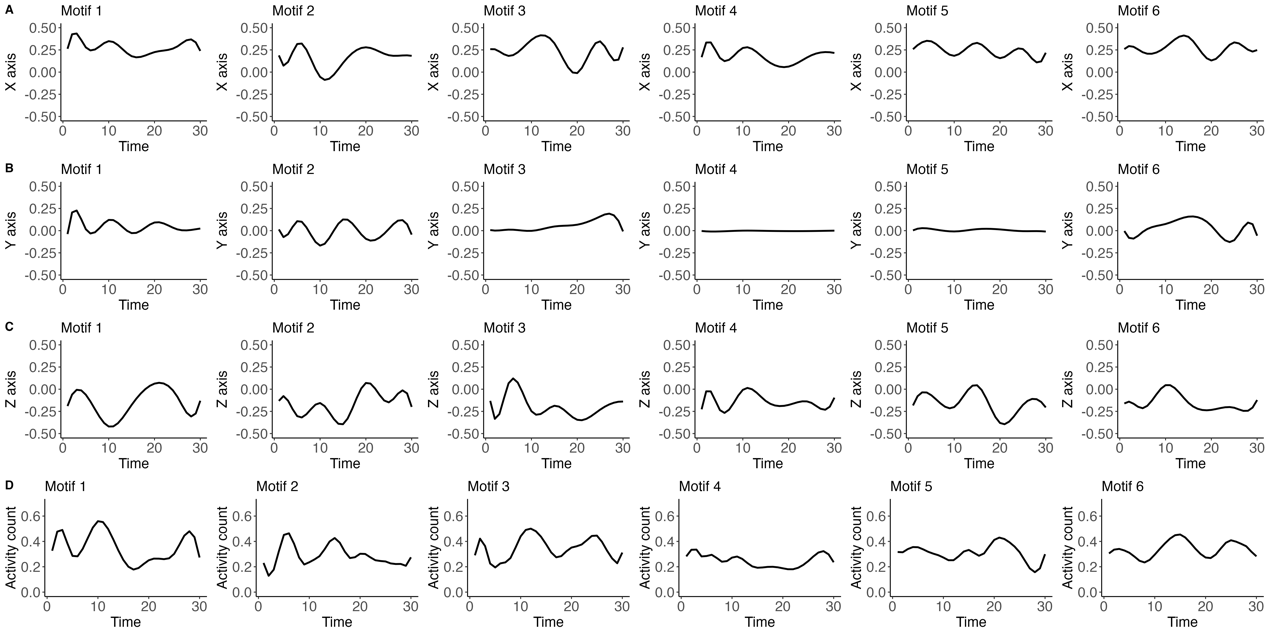


Figure S6. Visualization of mean activity functions (30 minutes) for each individual representing different motifs (6 motifs) obtained by the elastic distance-based motif clustering algorithm in the NHANES study. The mean activity function of the triaxial accelerometer is presented in (A) the X-axis, (B) the Y-axis, (C) the Z-axis, and (D) the combined magnitude of activity, calculated as $\sqrt{{x(t)}^{2}+{y(t)}^{2}+z\left( t \right)^{2}}$


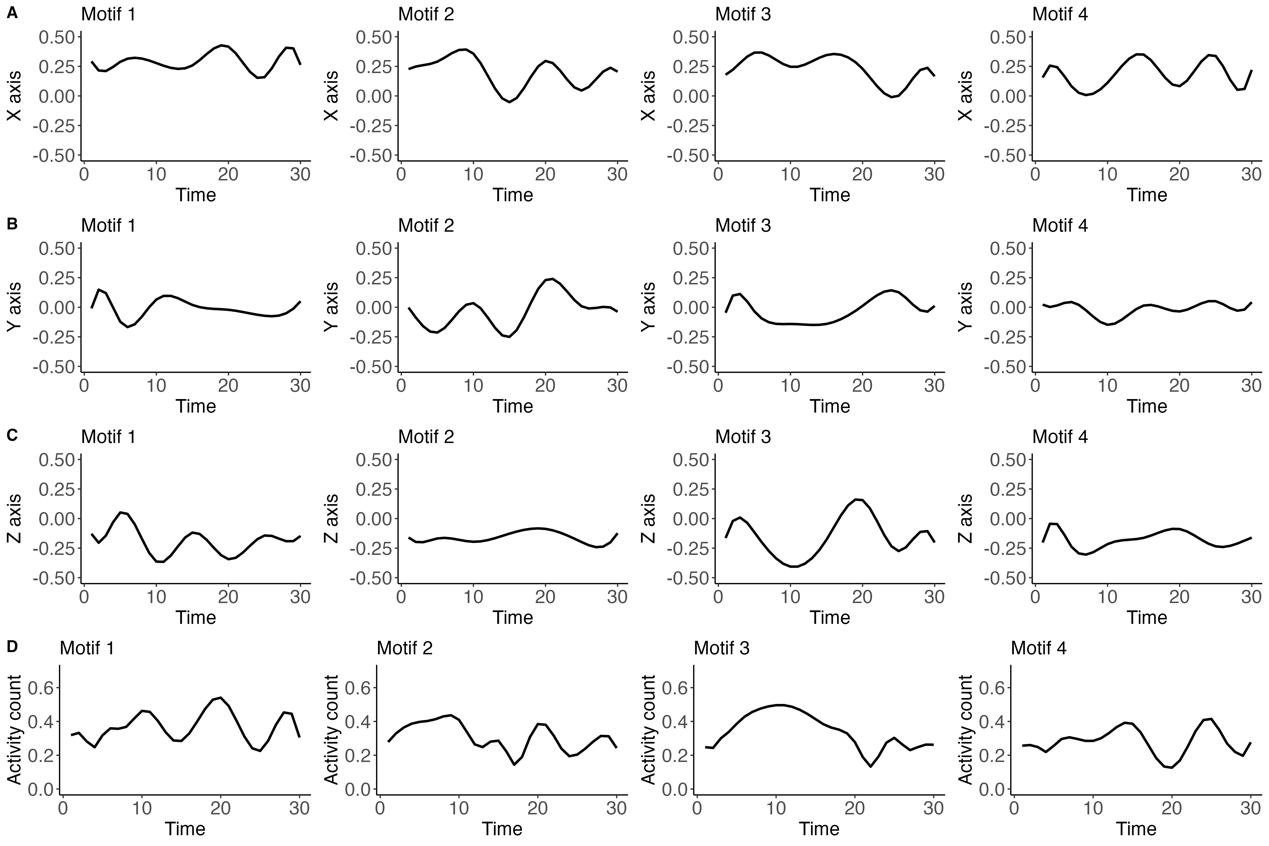


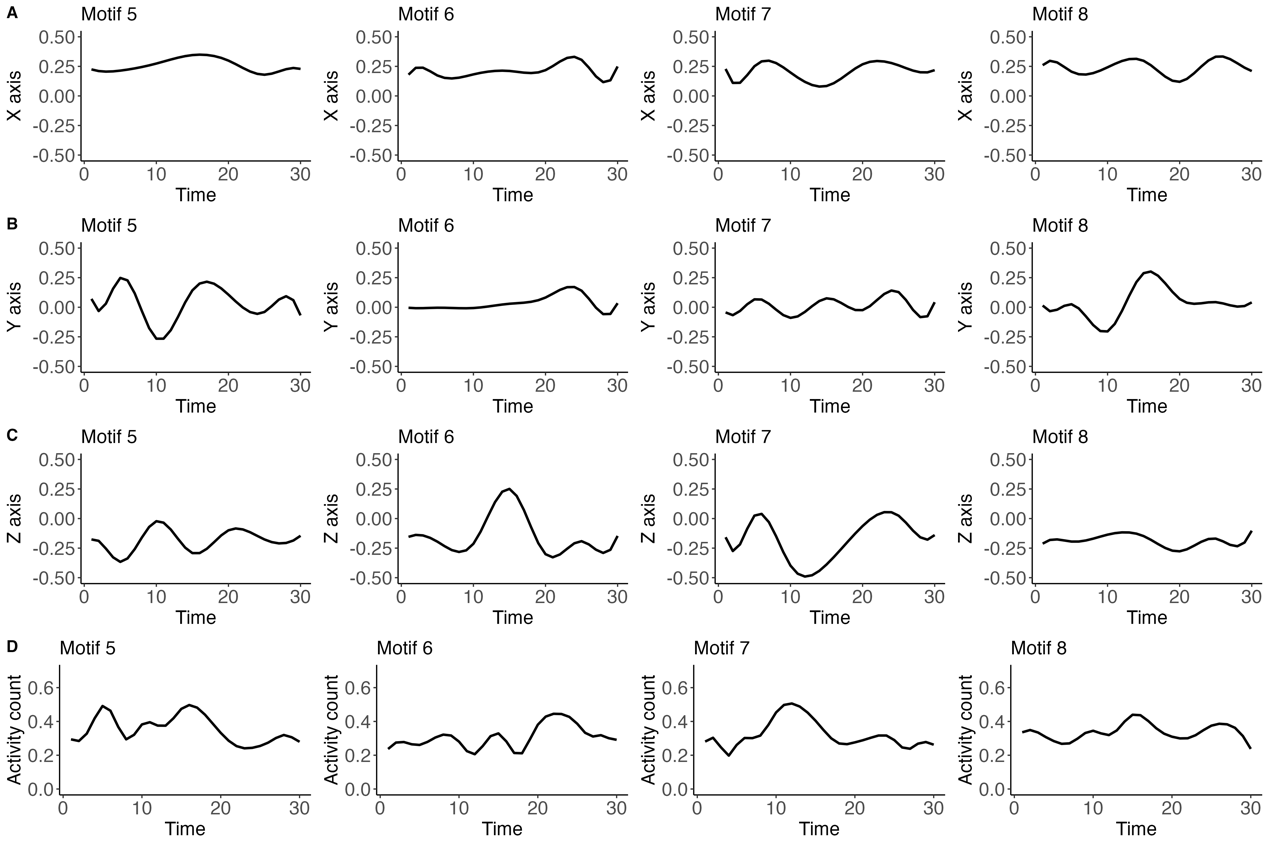


Figure S7. Visualization of mean activity functions (30 minutes) for each individual representing different motifs (8 motifs) obtained by the elastic distance-based motif clustering algorithm in the NHANES study. The mean activity function of the triaxial accelerometer is presented in (A) the X-axis, (B) the Y-axis, (C) the Z-axis, and (D) the combined magnitude of activity, calculated as $\sqrt{{x(t)}^{2}+{y(t)}^{2}+z\left( t \right)^{2}}$


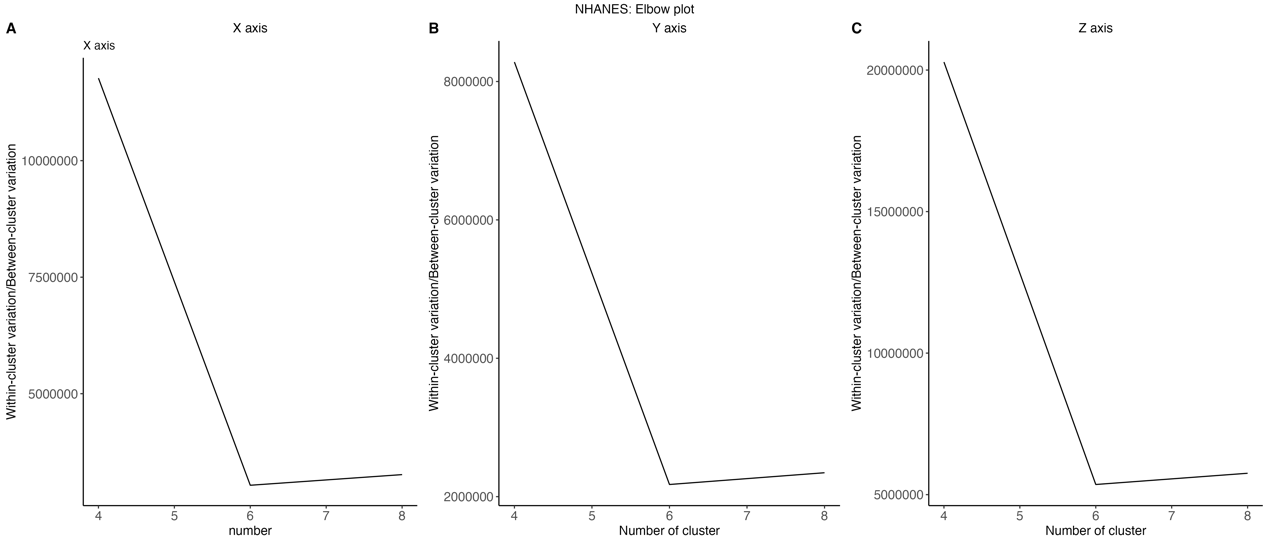


Figure S8. Elbow plot of the within-cluster variation to between-cluster variation ratio for (A) X axis (B) Y axis (C) Z axis in the NHANES study.

**Application 2: Two Mental Health study**

1. **Investigating Digital Biomarkers**


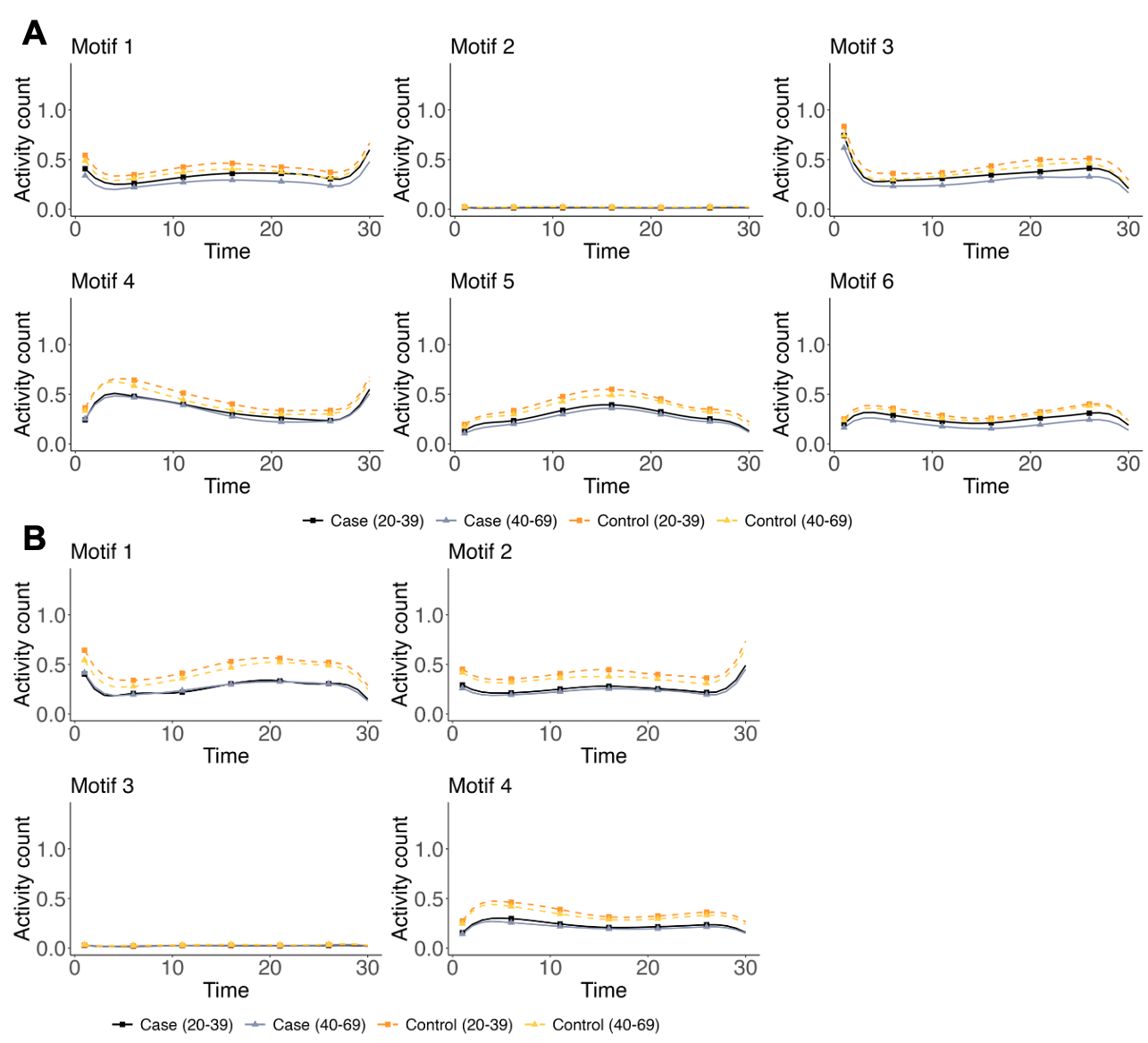
Figure S9. Visualization of the mean activity functions for different age groups representing different motifs obtained by the elastic distance-based motif clustering algorithm in the (A) Depresjon study and (B) PSYKOSE study


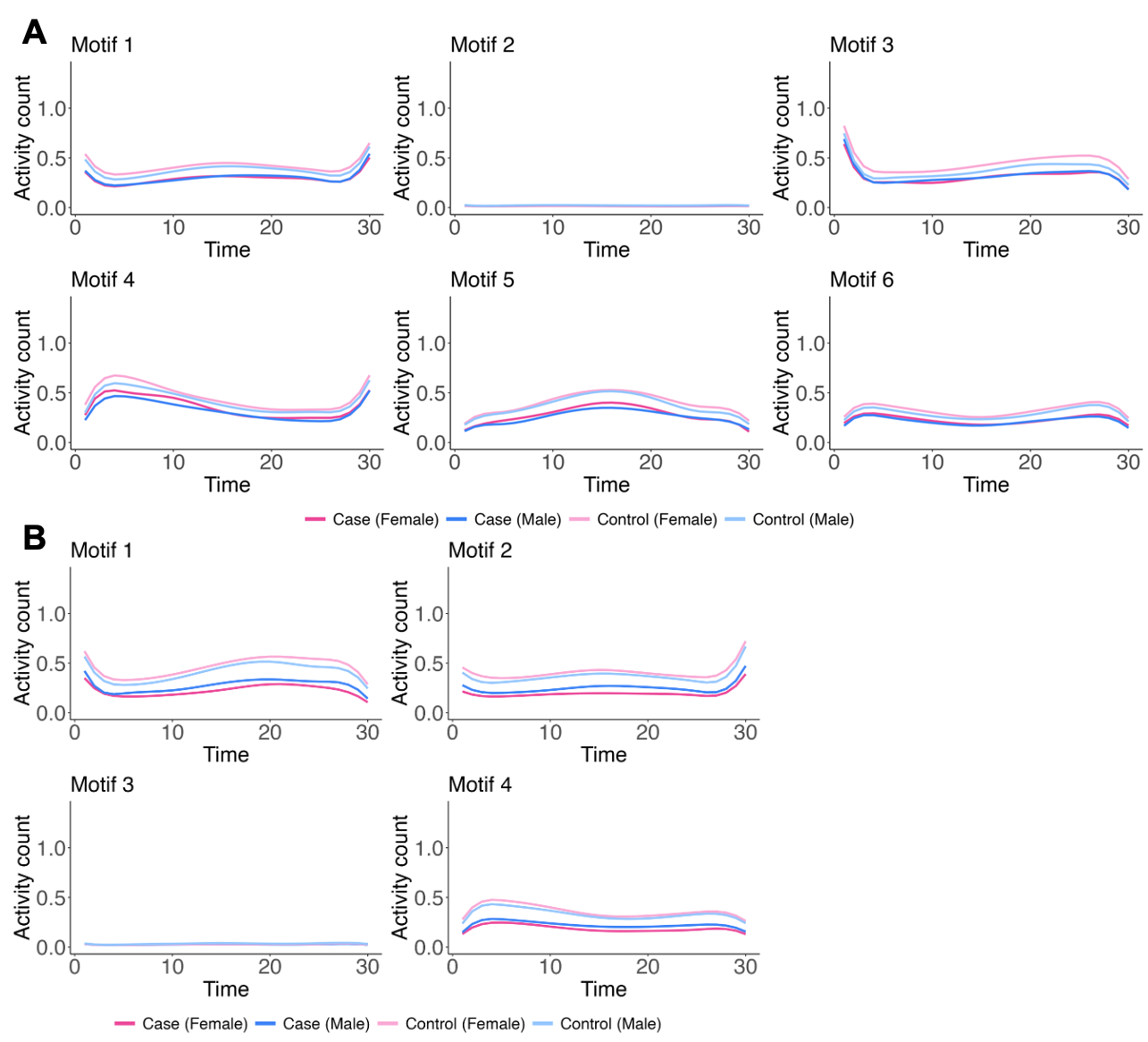
Figure S10. Visualization of mean activity functions for different gender groups representing different motifs obtained by the elastic distance-based motif clustering algorithm in the (A) Depresjon study and (B) PSYKOSE study


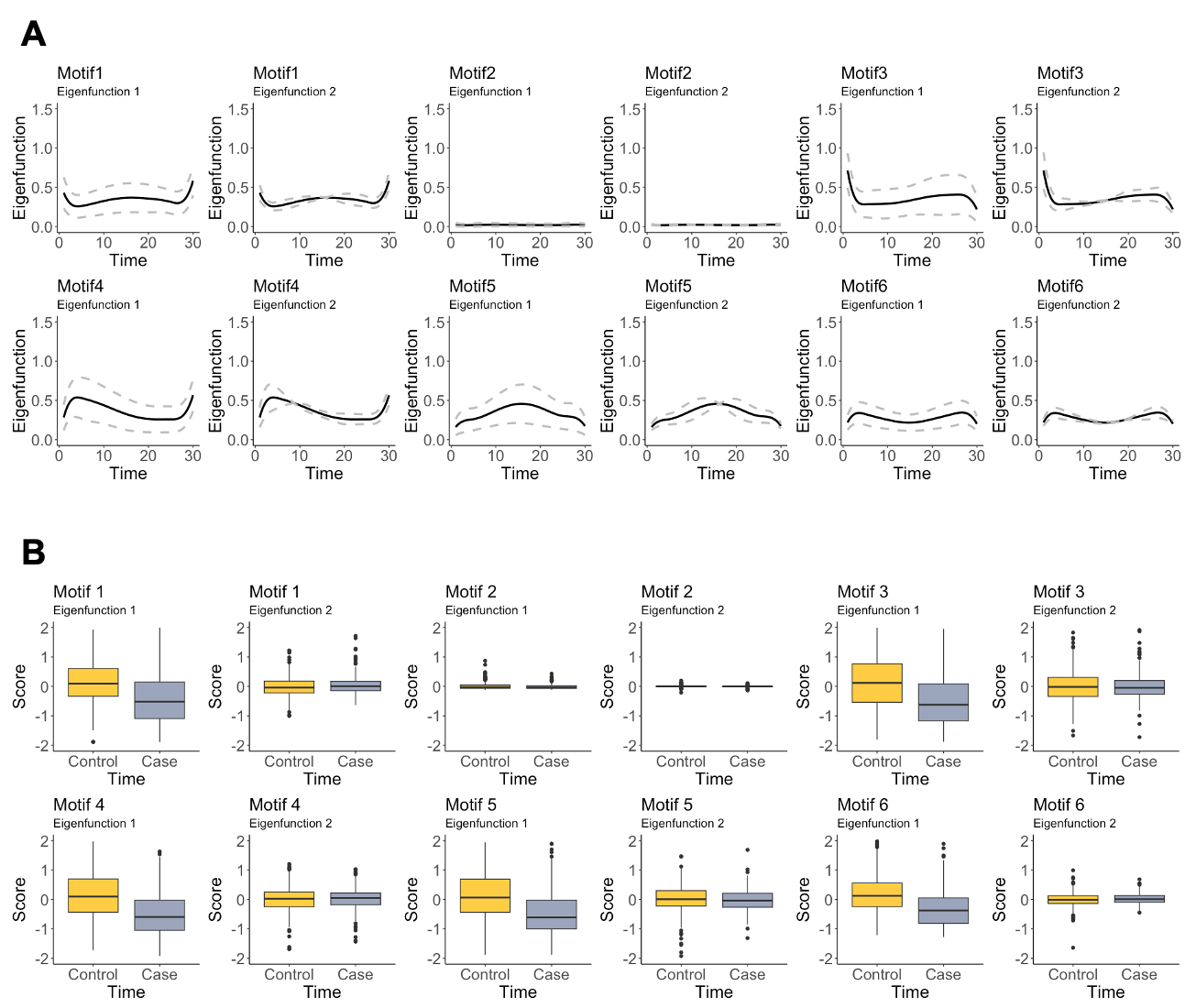


Figure S11. Visualization of the (A) eigenfunctions and (B) FPC scores corresponding to FPC1 and FPC2 for various motifs derived from the Depresjon study


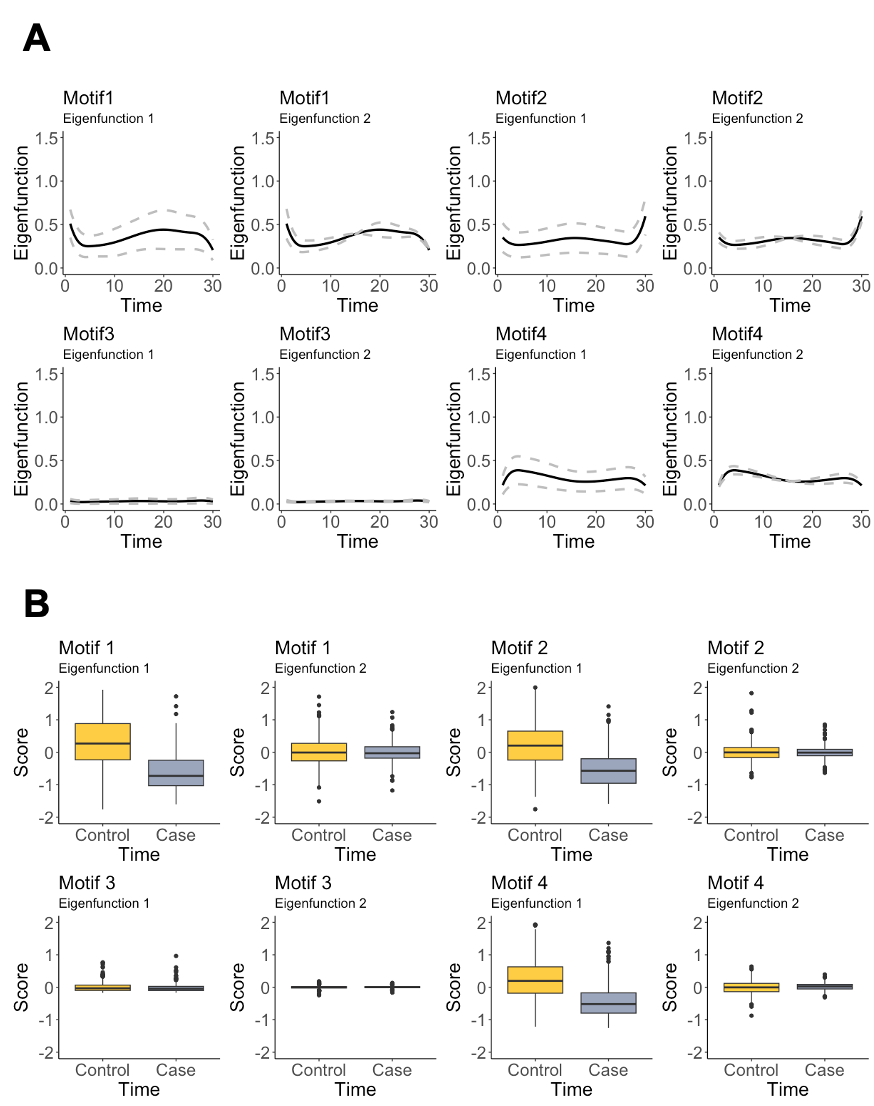


Figure S12. Visualization of the (A) eigenfunctions and (B) FPC scores corresponding to FPC1 and FPC2 for various motifs derived from the PSYKOSE study

Table S2. Baseline characteristics of each motif in Deprejson study

|  | Motif 1 (n=5255) | Motif 2 (n=11177) | Motif 3 (n=2981) | Motif 4 (n=3232) | Motif 5 (n=4059) | Motif 6 (n=8768) |
| --- | --- | --- | --- | --- | --- | --- |
| Type (n,%) |  |  |  |  |  |  |
| Control | 3346 (63.67) | 5906 (52.84) | 1877 (62.97) | 2076 (64.23) | 2437 (60.04) | 5142 (58.65) |
| Case | 1909 (36.33) | 5271 (47.16) | 1104 (37.03) | 1156 (35.77) | 1622 (39.96) | 3626 (41.35) |
| Gender (n,%) |  |  |  |  |  |  |
| Female | 2963 (56.38) | 6319 (56.54) | 1668 (55.95) | 1846 (57.12) | 2261 (55.7) | 4767 (54.37) |
| Male | 2292 (43.62) | 4858 (43.46) | 1313 (44.05) | 1386 (42.88) | 1798 (44.3) | 4001 (45.63) |
| Age (n,%) |  |  |  |  |  |  |
| 20-39 | 2584 (49.17) | 5327 (47.66) | 1541 (51.69) | 1640 (50.74) | 1955 (48.16) | 4233 (48.28) |
| 40-69 | 2671 (50.83) | 5850 (52.34) | 1440 (48.31) | 1592 (49.26) | 2104 (51.84) | 4535 (51.72) |
| Time (n,%) |  |  |  |  |  |  |
| 0:00-5:59 | 476 (9.06) | 5682 (50.84) | 307 (10.3) | 215 (6.65) | 620 (15.27) | 1568 (17.88) |
| 6:00-11:59 | 1382 (26.3) | 2670 (23.89) | 683 (22.91) | 837 (25.9) | 1029 (25.35) | 2267 (25.86) |
| 12:00-17:59 | 1827 (34.77) | 1098 (9.82) | 1077 (36.13) | 1146 (35.46) | 1202 (29.61) | 2518 (28.72) |
| 18:00-23:59 | 1570 (29.88) | 1727 (15.45) | 914 (30.66) | 1034 (31.99) | 1208 (29.76) | 2415 (27.54) |

Table S3. Distribution of motifs across time intervals and diseases in the Deprejson study.

|  | Depression (Case) | Healthy people (Control) |
| --- | --- | --- |
| **Motif 1 (N=5255)** | **1909** | **3346** |
| Time (n,%) |  |  |
| 0:00-5:59 | 204 (10.69) | 272 (8.13) |
| 6:00-11:59 | 466 (24.41) | 916 (27.38) |
| 12:00-17:59 | 657 (34.42) | 1170 (34.97) |
| 18:00-23:59 | 582 (30.49) | 988 (29.53) |
| **Motif 2 (N=11177)** | **5271** | **5906** |
| Time (n,%) |  |  |
| 0:00-5:59 | 2300 (43.63) | 3382 (57.26) |
| 6:00-11:59 | 1444 (27.40) | 1226 (20.76) |
| 12:00-17:59 | 619 (11.74) | 479 (8.11) |
| 18:00-23:59 | 908 (17.23) | 819 (13.87) |
| **Motif 3 (N=2981)** | **1104** | **1877** |
| Time (n,%) |  |  |
| 0:00-5:59 | 131 (11.90) | 186 (9.91) |
| 6:00-11:59 | 236 (21.40) | 447 (23.81) |
| 12:00-17:59 | 412 (37.30) | 665 (35.43) |
| 18:00-23:59 | 325 (29.40) | 589 (31.38) |
| **Motif 4 (N=3232)** | **1156** | **2076** |
| Time (n,%) |  |  |
| 0:00-5:59 | 95 (8.22) | 120 (5.78) |
| 6:00-11:59 | 264 (22.84) | 573 (27.60) |
| 12:00-17:59 | 442 (38.24) | 704 (33.91) |
| 18:00-23:59 | 355 (30.71) | 679 (32.71) |
| **Motif 5 (N=4059)** | **1622** | **2437** |
| Time (n,%) |  |  |
| 0:00-5:59 | 257 (15.84) | 363 (14.90) |
| 6:00-11:59 | 395 (24.35) | 634 (26.02) |
| 12:00-17:59 | 491 (30.27) | 711 (29.18) |
| 18:00-23:59 | 479 (29.53) | 729 (29.91) |
| **Motif 6 (N=8768)** | **3626** | **5142** |
| Time (n,%) |  |  |
| 0:00-5:59 | 685 (18.89) | 883 (17.17) |
| 6:00-11:59 | 867 (23.91) | 1400 (27.23) |
| 12:00-17:59 | 1051 (28.99) | 1467 (28.53) |
| 18:00-23:59 | 1023 (28.21) | 1392 (27.07) |

Table S4. Distribution of motifs across gender and diseases in the Deprejson study.

|  | Depression (Case) | | Healthy people (Control) | |
| --- | --- | --- | --- | --- |
|  | Female  (n=6720) | Male  (n=7968) | Female  (n=13104) | Male  (n=7680) |
| Motif 1 | 874 (13.01) | 1035 (12.99) | 2089 (15.94) | 1257 (16.37) |
| Motif 2 | 2520 (37.50) | 2751 (34.53) | 3799 (28.99) | 2107 (27.43) |
| Motif 3 | 476 (7.08) | 628 (7.88) | 1192 (9.10) | 685 (8.92) |
| Motif 4 | 542 (8.07) | 614 (7.71) | 1304 (9.95) | 772 (10.05) |
| Motif 5 | 739 (11.00) | 883 (11.08) | 1522 (11.61) | 915 (11.91) |
| Motif 6 | 1569 (23.35) | 2057 (25.82) | 3198 (24.40) | 1944 (25.31) |

Table S5. Distribution of motifs across age and diseases in the Deprejson study.

|  | Depression (Case) | | Healthy people (Control) | |
| --- | --- | --- | --- | --- |
|  | Age, 20-39  (n=5472) | Age, 40-69  (n=9216) | Age, 20-39  (n=11808) | Age, 40-69  (n=8976) |
| Motif 1 | 725 (13.25) | 1184 (12.85) | 1859 (15.74) | 1487 (16.57) |
| Motif 2 | 1863 (34.04) | 3408 (36.98) | 3464 (29.34) | 2442 (27.21) |
| Motif 3 | 453 (8.28) | 651 (7.06) | 1088 (9.21) | 789 (8.79) |
| Motif 4 | 459 (8.39) | 697 (7.56) | 1181 (10.00) | 895 (9.97) |
| Motif 5 | 619 (11.31) | 1003 (10.88) | 1336 (11.31) | 1101 (12.27) |
| Motif 6 | 1353 (24.73) | 2273 (24.66) | 2880 (24.39) | 2262 (25.20) |

Table S6. Baseline characteristics of each motif in PSYKOSE study.

|  | Motif 1 (n=4763) | Motif 2 (n=6638) | Motif 3 (n=12094) | Motif 4 (n=11497) |
| --- | --- | --- | --- | --- |
| Type (n,%) |  |  |  |  |
| Control | 3180 (66.76) | 4273 (64.37) | 6326 (52.31) | 7005 (60.93) |
| Case | 1583 (33.24) | 2365 (35.63) | 5768 (47.69) | 4492 (39.07) |
| Gender (n,%) |  |  |  |  |
| Female | 2186 (45.9) | 3001 (45.21) | 4911 (40.61) | 4926 (42.85) |
| Male | 2577 (54.1) | 3637 (54.79) | 7183 (59.39) | 6571 (57.15) |
| Age (n,%) |  |  |  |  |
| 20-39 | 2318 (48.67) | 3075 (46.32) | 5580 (46.14) | 5299 (46.09) |
| 40-69 | 2445 (51.33) | 3563 (53.68) | 6514 (53.86) | 6198 (53.91) |
| Time (n,%) |  |  |  |  |
| 0:00-5:59 | 394 (8.27) | 581 (8.75) | 6115 (50.56) | 1658 (14.42) |
| 6:00-11:59 | 1255 (26.35) | 1803 (27.16) | 2630 (21.75) | 3060 (26.62) |
| 12:00-17:59 | 1655 (34.75) | 2298 (34.62) | 1268 (10.48) | 3527 (30.68) |
| 18:00-23:59 | 1459 (30.63) | 1956 (29.47) | 2081 (17.21) | 3252 (28.29) |

Table S7. Distribution of motifs across time intervals and diseases in the PSYKOSE study.

|  | Schizophrenia (Case) | Healthy people (Control) |
| --- | --- | --- |
| **Motif 1 (n=4763)** | **1583** | **3180** |
| Time (n,%) |  |  |
| 0:00-5:59 | 121 (7.64) | 273 (8.58) |
| 6:00-11:59 | 435 (27.48) | 820 (25.79) |
| 12:00-17:59 | 565 (35.69) | 1090 (34.28) |
| 18:00-23:59 | 462 (29.19) | 997 (31.35) |
| **Motif 2 (N=6638)** | **2365** | **4273** |
| Time (n,%) |  |  |
| 0:00-5:59 | 201 (8.50) | 380 (8.89) |
| 6:00-11:59 | 634 (26.81) | 1169 (27.36) |
| 12:00-17:59 | 853 (36.07) | 1445 (33.82) |
| 18:00-23:59 | 677 (28.63) | 1279 (29.93) |
| **Motif 3 (N=12094)** | **5768** | **6326** |
| Time (n,%) |  |  |
| 0:00-5:59 | 2622 (45.46) | 3493 (55.22) |
| 6:00-11:59 | 1276 (22.12) | 1354 (21.40) |
| 12:00-17:59 | 716 (12.41) | 552 (8.73) |
| 18:00-23:59 | 1154 (20.01) | 927 (14.65) |
| **Motif 4 (N=11497)** | **4492** | **7005** |
| Time (n,%) |  |  |
| 0:00-5:59 | 608 (13.54) | 1050 (14.99) |
| 6:00-11:59 | 1207 (26.87) | 1853 (26.45) |
| 12:00-17:59 | 1418 (31.57) | 2109 (30.11) |
| 18:00-23:59 | 1259 (28.03) | 1993 (28.45) |

Table S8. Distribution of motifs across age and diseases in the PSYKOSE study.

|  | Schizophrenia | | Healthy people | |
| --- | --- | --- | --- | --- |
|  | Female (n=1920) | Male  (n=12288) | Female (n=13104) | Male  (n=7680) |
| Motif 1 (n,%) | 192 (10.00) | 1391 (11.32) | 1994 (15.22) | 1186 (15.44) |
| Motif 2 (n,%) | 285 (14.84) | 2080 (16.93) | 2716 (20.73) | 1557 (20.27) |
| Motif 3 (n,%) | 879 (45.78) | 4889 (39.78) | 4032 (30.77) | 2294 (29.87) |
| Motif 4 (n,%) | 564 (29.38) | 3928 (31.97) | 4362 (33.29) | 2643 (34.41) |

Table S9. Distribution of motifs across age and diseases in the PSYKOSE study.

|  | Schizophrenia | | Healthy people | |
| --- | --- | --- | --- | --- |
|  | Age, 20-39 (n=4464) | Age,40-69 (n=9744) | Age, 20-39 (n=11808) | Age, 40-69 (n=8976) |
| Motif 1 (n,%) | 528 (11.83) | 1055 (10.83) | 1790 (15.16) | 1390 (15.49) |
| Motif 2 (n,%) | 705 (15.79) | 1660 (17.04) | 2370 (20.07) | 1903 (21.20) |
| Motif 3 (n,%) | 1867 (41.82) | 3901 (40.03) | 3713 (31.44) | 2613 (29.11) |
| Motif 4 (n,%) | 1364 (30.56) | 3128 (32.10) | 3935 (33.32) | 3070 (34.20) |

1. **Apply multivariate functional principal component analysis (MFPCA) to**

To account for the potential correlations among scores across clusters, we performed a multivariate functional principal component analysis (MFPCA) using the ‘MFPCA’ package in R [1,2]. The results are detailed in the Supplementary Materials. While the eigenfunctions from MFPCA (Figure S13 and S15) were similar to those from individual FPCA (Figure S11(A) and S12(A)), MFPCA did not yield cluster-specific FPC scores. This limitation prevented us from achieving our primary objective of developing unique digital biomarkers for each identified motif through clustering.

**
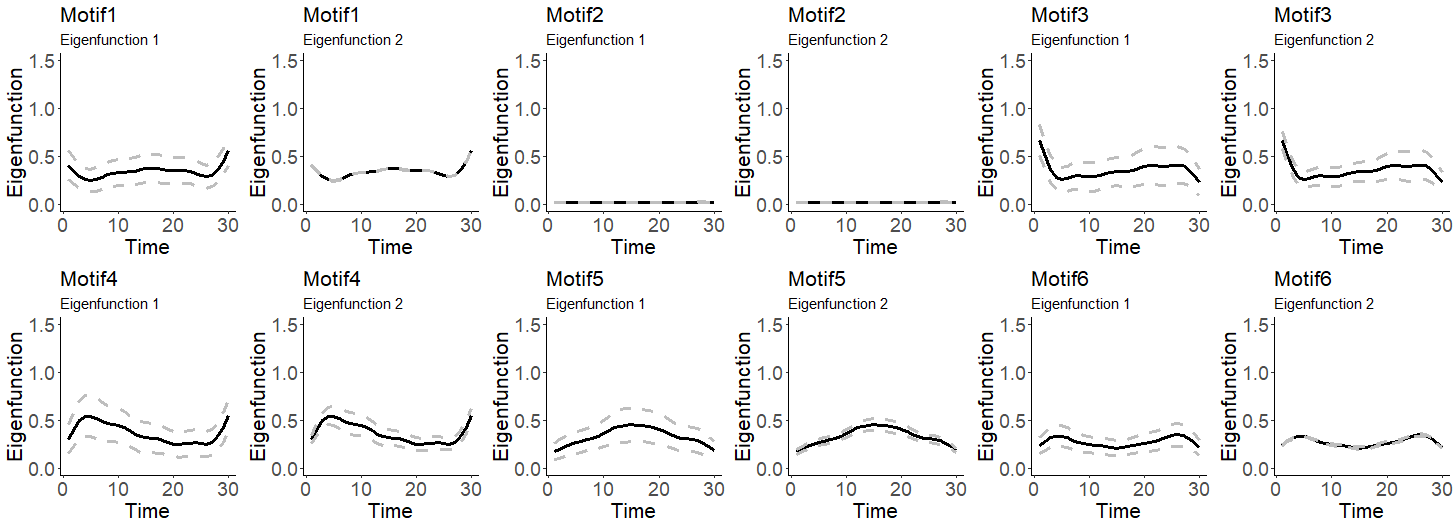
**

Figure S13. The eigenfunctions from MFPCA in the Deprejson study.

**
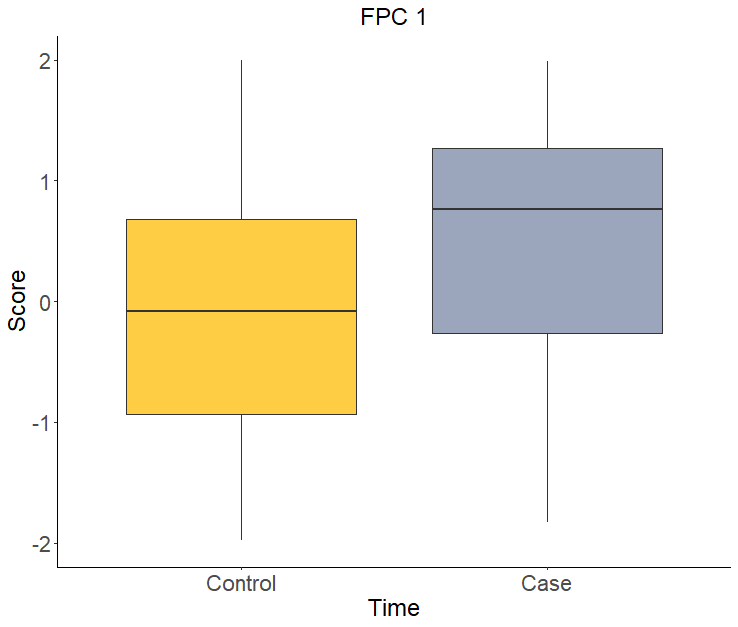

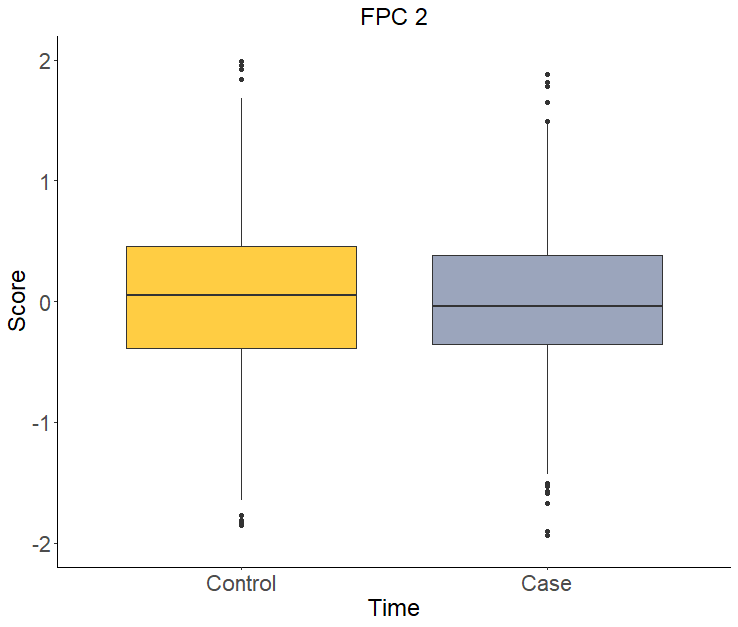
**

Figure S14. The score of FPC1 and FPC2 from MFPCA in the Deprejson study.

**
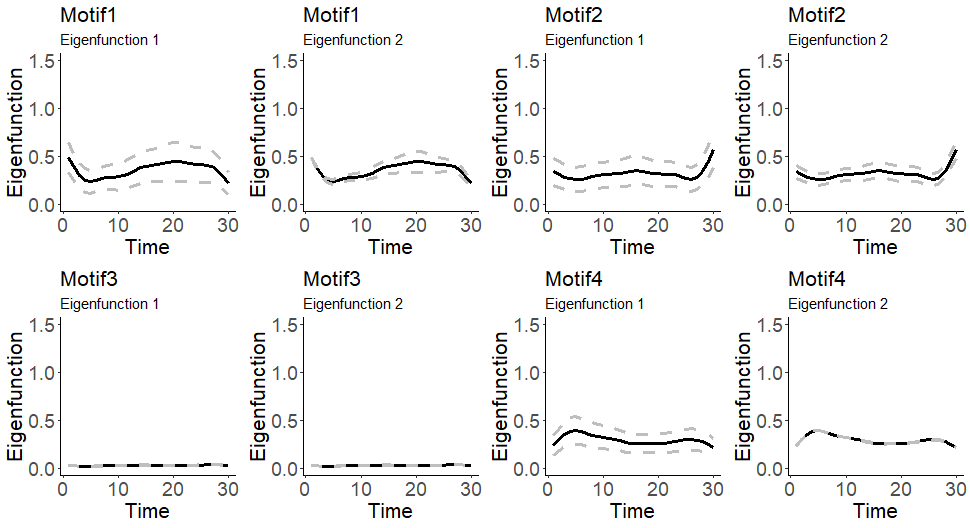
**

Figure S15. The eigenfunctions from MFPCA in the PSYKOSE study.

**
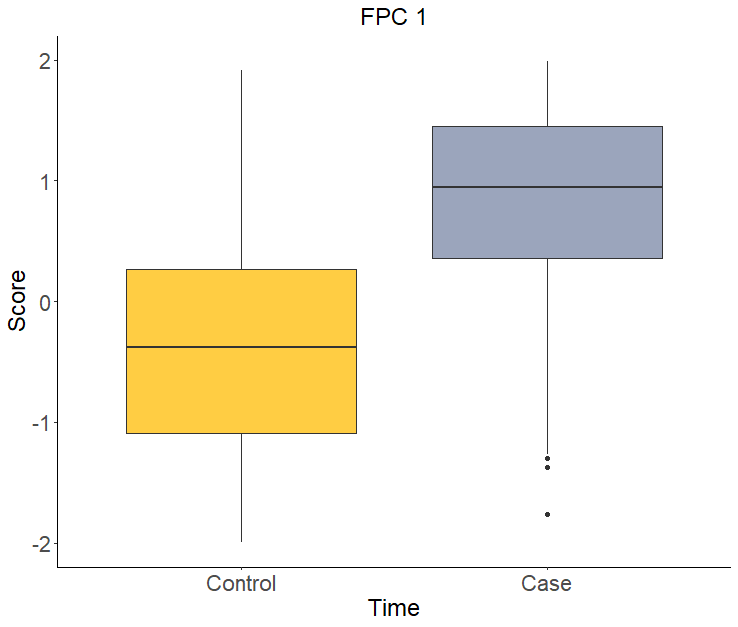
** **
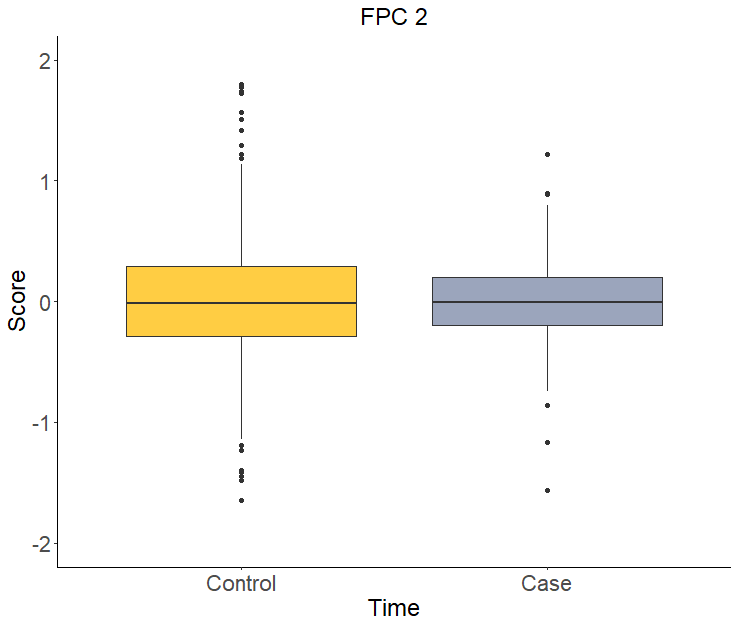
**

Figure S16. The score of FPC1 and FPC2 from MFPCA in the PSYKOSE study.

**Reference**

[1] Happ-Kurz C (2022). MFPCA: Multivariate Functional Principal Component Analysis for Data Observed on Different Dimensional Domains. R package version 1.3-10, https://github.com/ClaraHapp/MFPCA.

[2] Happ C, Greven S (2018). “Multivariate Functional Principal Component Analysis for Data Observed on Different (Dimensional) Domains.” *Journal of the American Statistical Association*. 113, 649-659. doi:10.1080/01621459.2016.1273115

1. **Activity patterns identified by the elastic distance-based motif clustering algorithm with different** $\boldsymbol{k}$ **and time windows**


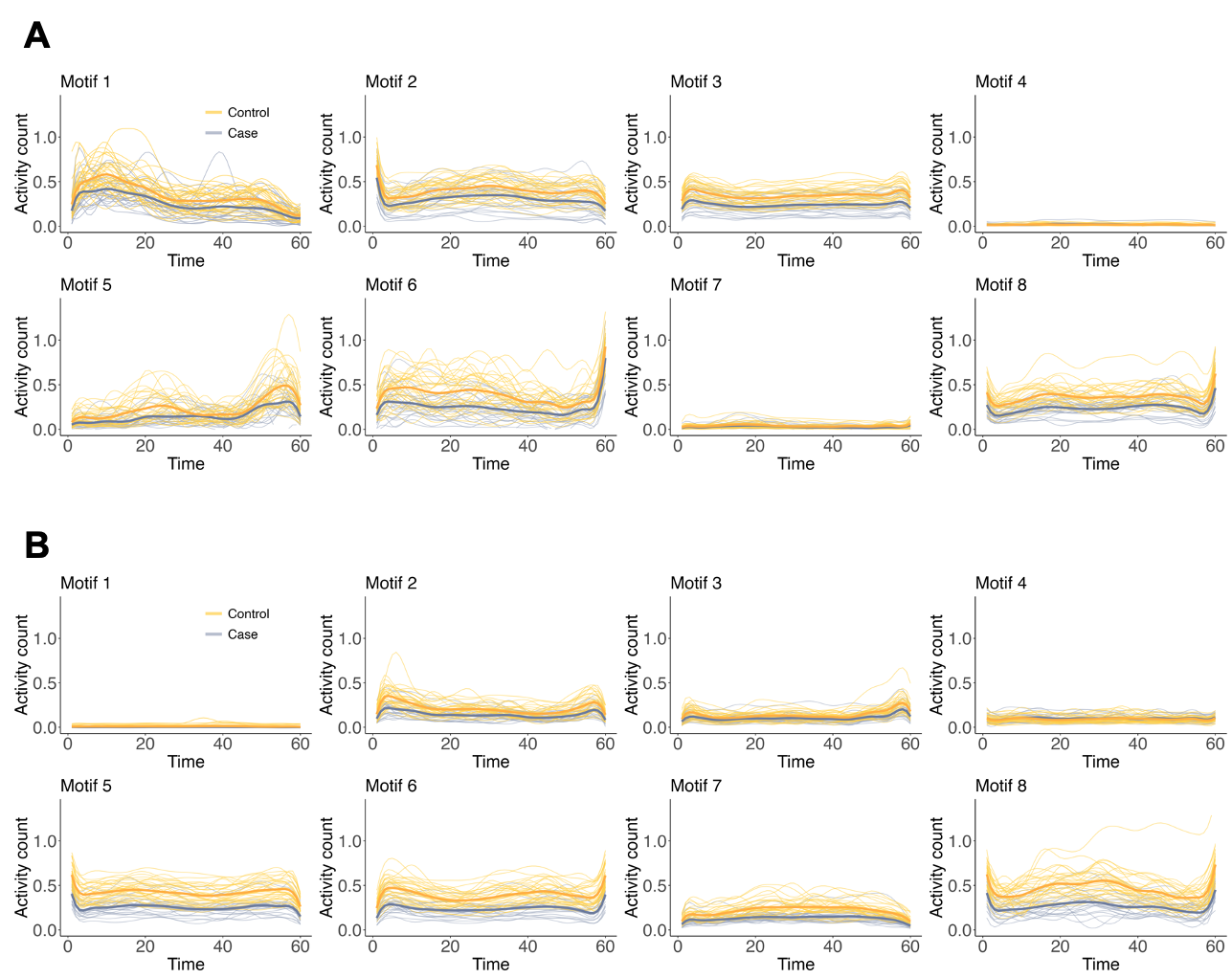


Figure S17. Visualization of mean activity functions (1 hour) for each individual representing different motifs obtained by the elastic distance-based motif clustering algorithm in the (A) Depresjon study and (B) PSYKOSE study


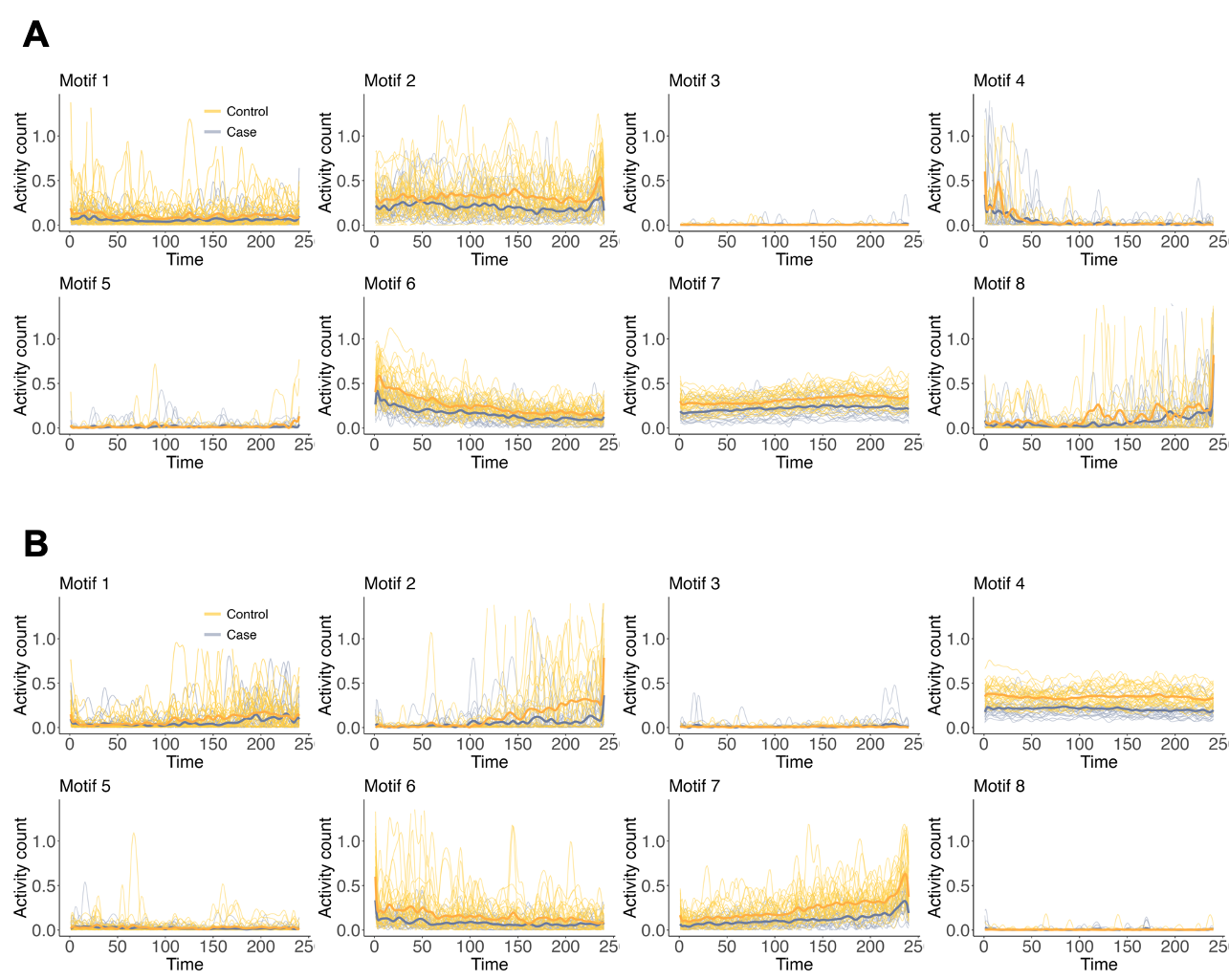


Figure S18. Visualization of mean activity function (4 hours) for each individual representing different motifs obtained by the elastic distance-based motif clustering algorithm in the (A) Depresjon study and (B) PSYKOSE study


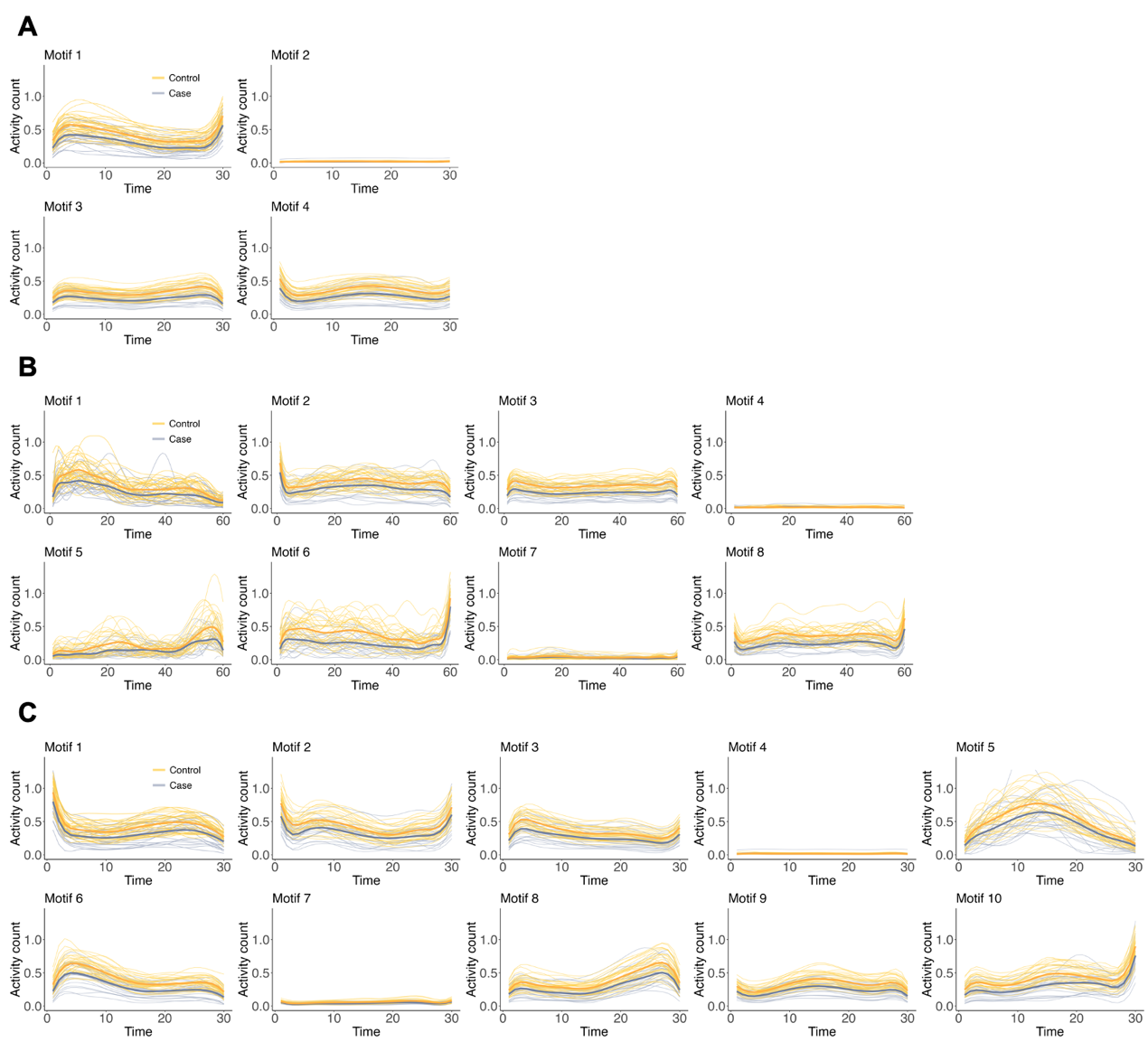


Figure S19. Visualization of mean activity function (30 minutes) for each individual representing different motifs obtained by the elastic distance-based motif clustering algorithm in the Depresjon study (A) 4 motifs (B) 8 motifs (C) 10 motifs


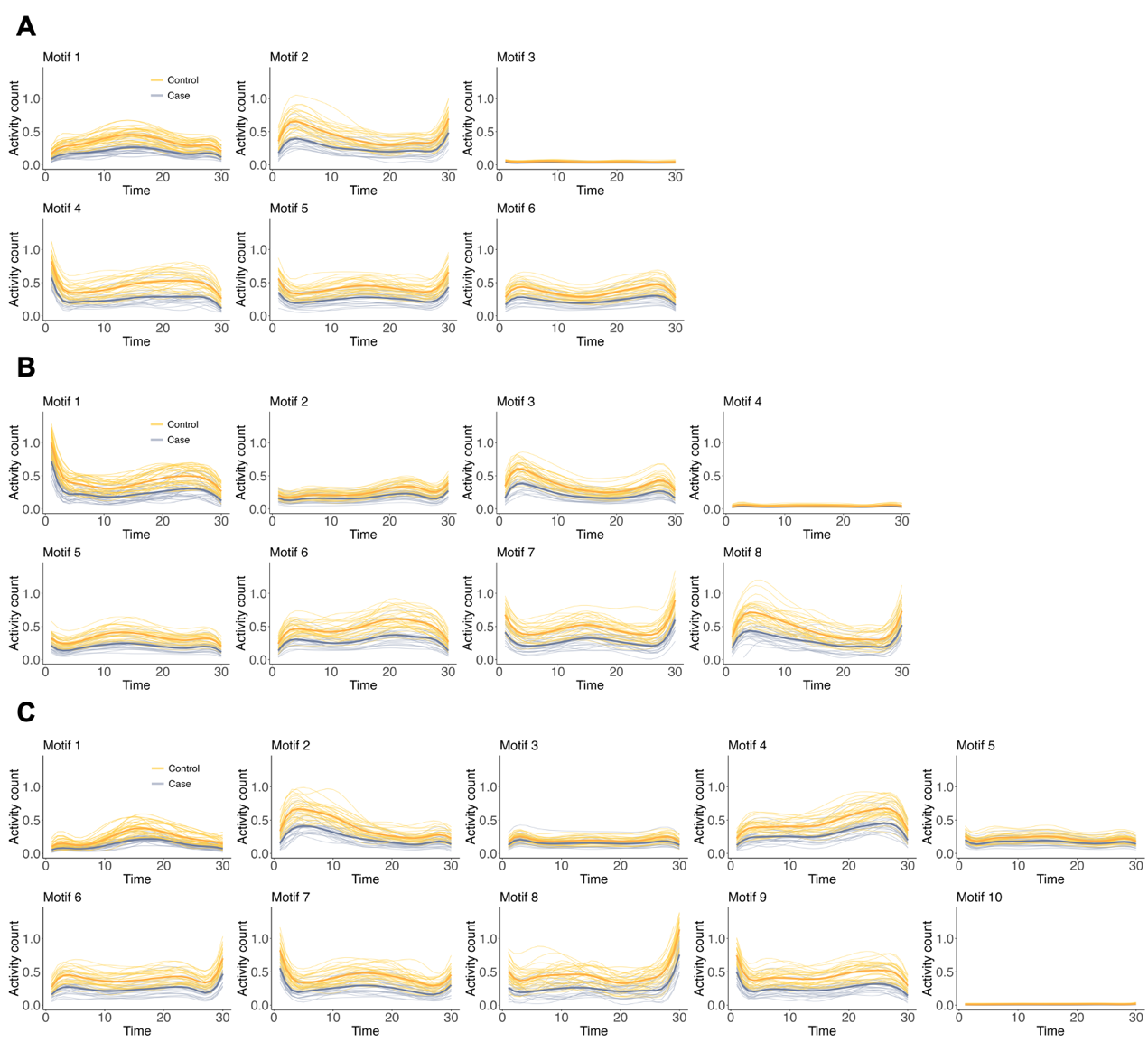


Figure S20. Visualization of the mean activity function (30 minutes) for each individual representing different motifs obtained by the elastic distance-based motif clustering algorithm in the PSYKOSE study (A) 6 motifs (B) 8 motifs (C) 10 motifs


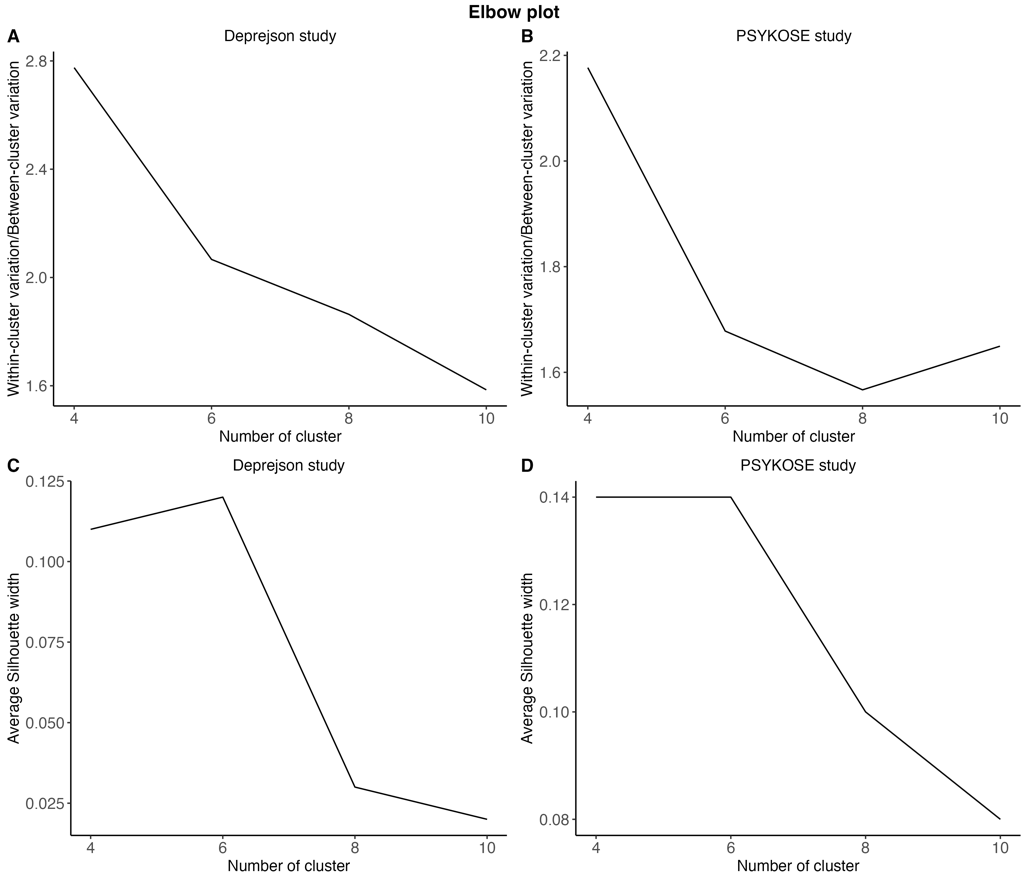


Figure S21. Elbow plot of the within-cluster variation to between-cluster variation ratio for the (A) Depresjon study (B) PSYKOSE study; Elbow plot of the average Silhouette width for the (C) Depresjon study (D) PSYKOSE study.

Table S10. Performance of classification model for mental health in the two application studies**.**

|  | **Accuracy** | | | **Sensitivity** | | **Specificity** |
| --- | --- | --- | --- | --- | --- | --- |
| **Depresjon study** | | | | | | |
| **30 mins: 8 digital biomarkers (4 motifs, 2 FPCs per motif) + no demographics** | | | | | | |
| Naïve Bayes | 0.67 | | | 0.70 | | 0.66 |
| SVM | 0.67 | | | 0.39 | | 0.88 |
| Logistic regression (Lasso) | 0.67 | | | 0.52 | | 0.78 |
| Decision Tree | 0.62 | | | 0.26 | | 0.88 |
| Random forests | 0.71 | | | 0.43 | | 0.91 |
| **30 mins: 8 digital biomarkers (4 motifs, 2 FPCs per motif) + demographics** | | | | | | |
| Naïve Bayes | 0.62 | | | 0.65 | | 0.59 |
| SVM | 0.73 | | | 0.48 | | 0.91 |
| Logistic regression (Lasso) | 0.62 | | | 0.48 | | 0.72 |
| Decision Tree | 0.62 | | | 0.26 | | 0.88 |
| Random forests | 0.75 | | | 0.52 | | 0.91 |
| **30 mins: 16 digital biomarkers (8 motifs, 2 FPCs per motif) + no demographics** | | | | | | |
| Naïve Bayes | 0.69 | | | 0.70 | | 0.69 |
| SVM | 0.69 | | | 0.39 | | 0.91 |
| Logistic regression (Lasso) | 0.67 | | | 0.52 | | 0.78 |
| Decision Tree | 0.73 | | | 0.48 | | 0.91 |
| Random forests | 0.69 | | | 0.43 | | 0.88 |
| **30 mins: 16 digital biomarkers (8 motifs, 2 FPCs per motif) + demographics** | | | | | | |
| Naïve Bayes | 0.67 | | | 0.70 | | 0.66 |
| SVM | 0.75 | | | 0.52 | | 0.91 |
| Logistic regression (Lasso) | 0.69 | | | 0.52 | | 0.81 |
| Decision Tree | 0.67 | | | 0.35 | | 0.91 |
| Random forests | 0.76 | | | 0.52 | | 0.94 |
| **30 mins: 20 digital biomarkers (10 motifs, 2 FPCs per motif) + no demographics** | | | | | | |
| Naïve Bayes | 0.62 | | | 0.65 | | 0.59 |
| SVM | 0.65 | | | 0.57 | | 0.88 |
| Logistic regression (Lasso) | 0.69 | | | 0.22 | | 0.78 |
| Decision Tree | 0.64 | | | 0.30 | | 0.94 |
| Random forests | 0.71 | | | 0.39 | | 0.94 |
| **30 mins: 20 digital biomarkers (10 motifs, 2 FPCs per motif) + demographics** | | | | | | |
| Naïve Bayes | 0.62 | | | 0.65 | | 0.59 |
| SVM | 0.71 | | | 0.48 | | 0.88 |
| Logistic regression (Lasso) | 0.65 | | | 0.52 | | 0.75 |
| Decision Tree | 0.64 | | | 0.26 | | 0.91 |
| Random forests | 0.71 | | | 0.43 | | 0.94 |
| **1 hour: 16 digital biomarkers (8 motifs, 2 FPCs per motif) + no demographics** | | | | | | |
| Naïve Bayes | 0.65 | | | 0.74 | | 0.59 |
| SVM | 0.73 | | | 0.48 | | 0.91 |
| Logistic regression (Lasso) | 0.71 | | | 0.57 | | 0.81 |
| Decision Tree | 0.71 | | | 0.43 | | 0.91 |
| Random forests | 0.69 | | | 0.43 | | 0.88 |
| **1 hour: 16 digital biomarkers (8 motifs, 2 FPCs per motif) + demographics** | | | | | | |
| Naïve Bayes | 0.64 | | | 0.70 | | 0.59 |
| SVM | 0.73 | | | 0.52 | | 0.88 |
| Logistic regression (Lasso) | 0.69 | | | 0.57 | | 0.78 |
| Decision Tree | 0.73 | | | 0.43 | | 0.94 |
| Random forests | 0.75 | | | 0.52 | | 0.91 |
| **4 hours: 16 digital biomarkers (8 motifs, 2 FPCs per motif) + no demographics** | | | | | | |
| Naïve Bayes | 0.53 | | | 0.96 | | 0.22 |
| SVM | 0.75 | | | 0.48 | | 0.94 |
| Logistic regression (Lasso) | 0.64 | | | 0.43 | | 0.78 |
| Decision Tree | 0.73 | | | 0.48 | | 0.91 |
| Random forests | 0.73 | | | 0.49 | | 0.91 |
| 4 **hours: 16 digital biomarkers (8 motifs, 2 FPCs per motif) + demographics** | | | | | | |
| Naïve Bayes | 0.55 | | | 0.91 | | 0.28 |
| SVM | 0.73 | | | 0.52 | | 0.88 |
| Logistic regression (Lasso) | 0.56 | | | 0.52 | | 0.59 |
| Decision Tree | 0.69 | | | 0.48 | | 0.88 |
| Random forests | 0.76 | | | 0.52 | | 0.94 |
| **PSYKOSE study** |  | | |  | |  |
| **30 mins: 12 digital biomarkers (6 motifs, 2 FPCs per motif) + no demographics** | | | | | | |
| Naïve Bayes | | 0.81 | 0.91 | | 0.75 | |
| SVM | | 0.85 | 0.77 | | 0.91 | |
| Logistic regression (Lasso) | | 0.80 | 0.77 | | 0.81 | |
| Decision Tree | | 0.83 | 0.68 | | 0.94 | |
| Random forests | | 0.87 | 0.77 | | 0.94 | |
| **30 mins: 12 digital biomarkers (6 motifs, 2 FPCs per motif) + demographics** | | | | | | |
| Naïve Bayes | | 0.81 | 0.86 | | 0.78 | |
| SVM | | 0.83 | 0.73 | | 0.91 | |
| Logistic regression (Lasso) | | 0.81 | 0.82 | | 0.81 | |
| Decision Tree | | 0.72 | 0.50 | | 0.88 | |
| Random forests | | 0.85 | 0.77 | | 0.91 | |
| **30 mins: 16 digital biomarkers (8 motifs, 2 FPCs per motif) + no demographics** | | | | | | |
| Naïve Bayes | | 0.81 | 0.86 | | 0.78 | |
| SVM | | 0.83 | 0.77 | | 0.88 | |
| Logistic regression (Lasso) | | 0.85 | 0.77 | | 0.91 | |
| Decision Tree | | 0.76 | 0.55 | | 0.91 | |
| Random forests | | 0.85 | 0.77 | | 0.91 | |
| **30 mins: 16 digital biomarkers (8 motifs, 2 FPCs per motif) + demographics** | | | | | | |
| Naïve Bayes | | 0.83 | 0.91 | | 0.78 | |
| SVM | | 0.87 | 0.86 | | 0.88 | |
| Logistic regression (Lasso) | | 0.83 | 0.82 | | 0.84 | |
| Decision Tree | | 0.74 | 0.55 | | 0.88 | |
| Random forests | | 0.89 | 0.77 | | 0.97 | |
| **30 mins: 20 digital biomarkers (10 motifs, 2 FPCs per motif) + no demographics** | | | | | | |
| Naïve Bayes | | 0.78 | 0.86 | | 0.72 | |
| SVM | | 0.89 | 0.82 | | 0.94 | |
| Logistic regression (Lasso) | | 0.85 | 0.86 | | 0.84 | |
| Decision Tree | | 0.80 | 0.59 | | 0.94 | |
| Random forests | | 0.87 | 0.77 | | 0.94 | |
| **30 mins: 20 digital biomarkers (10 motifs, 2 FPCs per motif) + demographics** | | | | | | |
| Naïve Bayes | | 0.80 | 0.86 | | 0.75 | |
| SVM | | 0.81 | 0.77 | | 0.84 | |
| Logistic regression (Lasso) | | 0.83 | 0.82 | | 0.84 | |
| Decision Tree | | 0.78 | 0.73 | | 0.81 | |
| Random forests | | 0.85 | 0.73 | | 0.94 | |
| **1 hour: 16 digital biomarkers (8 motifs, 2 FPCs per motif) + no demographics** | | | | | | |
| Naïve Bayes | | 0.74 | 0.91 | | 0.62 | |
| SVM | | 0.87 | 0.82 | | 0.91 | |
| Logistic regression (Lasso) | | 0.83 | 0.77 | | 0.88 | |
| Decision Tree | | 0.87 | 0.73 | | 0.97 | |
| Random forests | | 0.81 | 0.73 | | 0.88 | |
| **1 hour: 16 digital biomarkers (8 motifs, 2 FPCs per motif) + demographics** | | | | | | |
| Naïve Bayes | | 0.72 | 0.91 | | 0.59 | |
| SVM | | 0.89 | 0.82 | | 0.94 | |
| Logistic regression (Lasso) | | 0.78 | 0.73 | | 0.81 | |
| Decision Tree | | 0.87 | 0.68 | | 1.00 | |
| Random forests | | 0.83 | 0.68 | | 0.94 | |
| **4 hours: 16 digital biomarkers (8 motifs, 2 FPCs per motif) + no demographics** | | | | | | |
| Naïve Bayes | | 0.76 | 0.95 | | 0.62 | |
| SVM | | 0.85 | 0.77 | | 0.91 | |
| Logistic regression (Lasso) | | 0.78 | 0.77 | | 0.78 | |
| Decision Tree | | 0.70 | 0.59 | | 0.78 | |
| Random forests | | 0.87 | 0.73 | | 0.97 | |
| **4 hours: 16 digital biomarkers (8 motifs, 2 FPCs per motif) + demographics** | | | | | | |
| Naïve Bayes | | 0.76 | 1.00 | | 0.59 | |
| SVM | | 0.87 | 0.82 | | 0.91 | |
| Logistic regression (Lasso) | | 0.80 | 0.77 | | 0.81 | |
| Decision Tree | | 0.74 | 0.55 | | 0.88 | |
| Random forests | | 0.89 | 0.77 | | 0.97 | |

Table S11. GLMM for mental health, including effect estimates and 95% confidence intervals, were conducted using the daily average for each subject as input in the two application studies.

| **Variable** | **Odd ratio** | **P-value** |
| --- | --- | --- |
| **Depresjon study** |  |  |
| Cluster 5 FPC 1 | 0.0975 (0.026, 0.373) | < 0.001 |
| **PSYKOSE study** |  |  |
| Cluster 1 FPC 1 | 0.03 (0.00428, 0.201) | < 0.001 |
